# Temporal Fluctuation in North East Baltic Sea Region Cattle Population Revealed by Mitochondrial and Y-Chromosomal DNA Analyses

**DOI:** 10.1371/journal.pone.0123821

**Published:** 2015-05-20

**Authors:** Marianna Niemi, Auli Bläuer, Terhi Iso-Touru, Janne Harjula, Veronica Nyström Edmark, Eve Rannamäe, Lembi Lõugas, Antti Sajantila, Kerstin Lidén, Jussi-Pekka Taavitsainen

**Affiliations:** 1 Biotechnology and Food Research, MTT Agrifood Research Finland, Jokioinen, Finland; 2 University of Helsinki, Department of Forensic Medicine, Helsinki, Finland; 3 Department of Archaeology, University of Turku, Turku, Finland; 4 Department of Zoology, Stockholm University, Stockholm, Sweden; 5 Institute of History and Archaeology, University of Tartu, Tartu, Estonia; 6 Institute of history, Tallinn University, Tallinn, Estonia; 7 Archaeological Research Laboratory, Stockholm University, Stockholm, Sweden; Kunming Institute of Zoology, Chinese Academy of Sciences, CHINA

## Abstract

**Background:**

Ancient DNA analysis offers a way to detect changes in populations over time. To date, most studies of ancient cattle have focused on their domestication in prehistory, while only a limited number of studies have analysed later periods. Conversely, the genetic structure of modern cattle populations is well known given the undertaking of several molecular and population genetic studies.

**Results:**

Bones and teeth from ancient cattle populations from the North-East Baltic Sea region dated to the Prehistoric (Late Bronze and Iron Age, 5 samples), Medieval (14), and Post-Medieval (26) periods were investigated by sequencing 667 base pairs (bp) from the mitochondrial DNA (mtDNA) and 155 bp of intron 19 in the Y-chromosomal *UTY* gene. Comparison of maternal (mtDNA haplotypes) genetic diversity in ancient cattle (45 samples) with modern cattle populations in Europe and Asia (2094 samples) revealed 30 ancient mtDNA haplotypes, 24 of which were shared with modern breeds, while 6 were unique to the ancient samples. Of seven Y-chromosomal sequences determined from ancient samples, six were Y2 and one Y1 haplotype. Combined data including Swedish samples from the same periods (64 samples) was compared with the occurrence of Y-chromosomal haplotypes in modern cattle (1614 samples).

**Conclusions:**

The diversity of haplogroups was highest in the Prehistoric samples, where many haplotypes were unique. The Medieval and Post-Medieval samples also show a high diversity with new haplotypes. Some of these haplotypes have become frequent in modern breeds in the Nordic Countries and North-Western Russia while other haplotypes have remained in only a few local breeds or seem to have been lost. A temporal shift in Y-chromosomal haplotypes from Y2 to Y1 was detected that corresponds with the appearance of new mtDNA haplotypes in the Medieval and Post-Medieval period. This suggests a replacement of the Prehistoric mtDNA and Y chromosomal haplotypes by new types of cattle.

## Introduction

Archaeological and mitochondrial DNA evidence indicate that cattle were domesticated from the auroch (*Bos primigenius*) [1–5], about 10,000 years ago in the Fertile Crescent [6]. From the Fertile Crescent, domestic cattle spread to South Eastern Europe around 8,800 Before Present (BP), to Central Europe around 7,000 BP, and to North Central Europe after 6,700 BP [7]. Domestic cattle reached southern Scandinavia by 6,000 BP [8], Estonia by 4,100 BP [7] and finally Finland in the northern Baltic Sea region by 3,000 BP [9]. The oldest radiocarbon dated remains of cattle in Finland date back to 3086 ± 30 BP [9].

Molecular analyses of mitochondrial DNA and the Y-chromosome can be used to trace bovine maternal and paternal lineages, respectively [10,11]. Variation in the hypervariable region of the mithochondrial D-loop defines the majority of taurine cattle, as well as some mitochondrial lineages of Near Eastern aurochs and many Italian aurochs [12], to belong to the T mega-haplogroup, including the haplogroups T, T1, T2, T3, and T4 [1,5,13–15]. A study of the whole mitochondrial DNA has suggested an additional haplogroup, T5, defined by sites outside of the D-loop region [15]. Three other haplogroups have been identified in taurine cattle, where the closest in phylogeny to haplogroup T is haplogroup Q [10], differing by one diagnostic SNP site in the hypervariable region (position 15953 in V00654) [15]. Haplogroup Q has been found at low frequency in modern South European cattle breeds [10,15]. The distribution of haplogroup Q has been hypothesised to indicate a parallel Near eastern origin for haplogroups T and Q, where Q represents a minor domesticated lineage [16]. Haplogroup P that has only been identified in northern and central European aurochs, and in a couple of scattered taurine samples, diverged from T and Q prior to their split [15,17]. The oldest diverging branch in the mtDNA phylogeny is the very rare haplogroup R that has only been identified in local Italian cattle breeds [16].

The genetic diversity of the T haplogroup is highest in the Near and Middle East cattle populations, where four haplogroups T, T1, T2, and T3 exist [1,14], indicating a Near Eastern origin of taurine cattle, which is also supported by nuclear marker analyses that show higher variability in the Near East than in other regions [1,18]. European domestic cattle carry the same four haplogroups as Near East cattle, but with T3 predominating in Europe at least from the Neolithic period onwards [11,14,17,19,20]. Haplogroup T1 is quite frequent across the Mediterranean countries [3,21], and predominant and almost fixed in Africa [14]. Haplogroup T4 derives from T3 and has thus far only been detected in Asian and Yakutian cattle from Russian Siberia [11]. The star-like patterns of the T3-centered haplotypes detected in modern and Neolithic European cattle populations have been suggested to result from post-domestic accumulation of mutations [14,19].

A north—south gradient of genetic diversity has been detected in modern European cattle (*Bos taurus*), [11,18], including the Y-chromosome [11]. A single nucleotide polymorphism in intron 19 of the *UTY* gene (UTY19) can be used to distinguish between the two Y-chromosomal haplotypes, Y1 and Y2 [22]. Whereas Y1 is the dominating haplotype in modern Western and Northern European breeds, haplotype Y2 dominates in South European breeds [22], with a clear dividing zone in central Europe [23]. Apart from the geographical variation, a temporal fluctuation in Y1 and Y2 haplotype frequencies has been detected, mainly from Swedish ancient bulls and aurochs, suggesting that variation in present-day frequencies of Y1 and Y2 haplotypes is likely due to recent demographic events [24].

The aim of this study was to explore temporal population variation by maternally and paternally inherited markers in cattle from the North East Baltic Sea region (N-EBSR), and to compare ancient populations with modern breeds. Haplotype data from 45 ancient mtDNA and 7 Y-chromosome samples was used together with contemporary data from 2094 mtDNA [10,11,15,16,20] and 1614 modern [22–24] and 71 ancient Y-chromosomes [24–26] samples. The data indicates clear changes in the N-EBSR cattle populations from late Bronze/Iron Age to modern times.

## Materials and Methods

### Ancient cattle bones

A total of 77 cattle bones were selected for aDNA analysis from different sites across Finland and Estonia and in the town of Vyborg in the Leningrad Region in north-western Russia (Fig A in S1 File). The samples for this study were from museum collections held at 1.) The National Board of Antiquities, 2.) Museum of Raisio (Harkko), 3.) The Museum Centre of Turku, 4.) Ålands Museum, 5.) Museum of Viljandi, 6.) Pärnu Museum, 7.) Saaremaa Museum, 8.) University of Turku, 9.) St. Petersburg, Institute for the Material Culture History, Russian Academy of Sciences, 10.) Tallinn University, and 11.) University of Tartu (Table A in S1 File). All necessary permits were obtained for the described study, which complied with all relevant regulations.

The samples from Vyborg derive from the Medieval and Post-Medieval periods, during which Vyborg was part of Finland. The earliest bones (2 samples) available for this study derive from the Late Bronze Age (700–500 BC) from the island of Saaremaa, Estonia. The rest of the Prehistoric samples dates to the Late Iron Age (800–1200 AD). To verify that each individual within one site and period was sampled only once, samples deriving from the same side of the animal were selected, or the size and age of the individual was used to separate individuals. Whenever possible, metacarpals were preferred as metacarpals are used to osteologically determine the sex of the animal [27,28]. From 77 samples initially selected, a total of 18 bones or teeth from the Prehistoric period (700 BC-1200 AD), 24 from the Medieval period (1200–1550 AD), and 34 from the Post-Medieval period (1550–1800 AD) were used for aDNA analyses. One sample that was radiocarbon dated as modern was omitted from further aDNA analysis. A total of 21 skeletal samples were radiocarbon-dated at the Laboratory of Chronology of the Finnish Museum of Natural History (LUOMUS), University of Helsinki (Table A in S1 File). Radiocarbon dated samples covered all bones and teeth from non-distinct cultural layers that were used for aDNA analyses.

### DNA markers and laboratory methods

To determine the mtDNA haplogroups T, T1, T2, T3, T4, and T5 [15], a combination of three fragments yielding 486 bp of sequence covering the mtDNA D-loop from position 16031 to 178 [GenBank: V00654] and a 181 bp sequence from the ND5 gene (position 12 911 to 13 091[GenBank: V00654]) were analysed. An additional 77 bp D-loop fragment (positions 15936–16012 in V00654), determining haplotype Q, was analysed from one sample (H01, BtTor4). As a Y-chromosomal haplotype marker, a 155 bp sequence from intron 19 in the *UTY* gene was analysed (the transversion G>T at position 423 in [GenBank: AY936543], defining haplotypes Y1 or Y2) [22]. DNA extraction [29], PCR methods and sequencing of PCR products were as described in [30]. Briefly, 0.2–0.5 ml of bone powder was suspended in 900 μl 0.5M EDTA, 100 μl 10M urea and 5 μl proteinase K (20 mg/ml), and incubated with constant shaking at 55°C overnight. DNA from the concentrated supernatant (Amicon-4 30K centrifugal filter units, Merck Millipore) was extracted with a QIAquick PCR Purification Kit (Qiagen, Sweden) according to manufacturer’s instructions. Approximately 5–10 μL of DNA extract was used in the PCR performed with the HotStarTaq DNA polymerase Kit (Qiagen, Sweden) with an inclusion of 0.4 mM dNTP, 0.2 μM of each primer and 0.25 units (U) of Uracil DNA Glycosylase (UNG, Sigma-Aldrich). The PCR program included initial steps of 37°C for 10 min and 95°C for 15 min followed by 55 three-step cycles of 94°C for 30s, AT°C for 40s and 72°C for 1 min and 10 min at 72°, where AT stands for a specific annealing temperature for each primer pair (Table B in S1 File). Primers and success rates of aDNA analyses (Text A in S1 File, Table B in S1 File) are provided in the Supporting Information.

### Authenticity of ancient cattle DNA

The authenticity of aDNA analyses was controlled in various steps of the laboratory work-flow and the analyses were repeated in independent ancient DNA laboratories. All 45 ancient samples included in the statistical analyses were extracted at least twice (MTT Agrifood Research Finland, Jokioinen, Finland, Stockholm University, Stockholm, Sweden and Department of Forensic Medicine, University of Helsinki, Helsinki, Finland).

Each participating ancient DNA laboratory followed general guidelines for ancient DNA work such as separate space for sample preparation and ancient DNA work, separate pre- and post-PCR areas, air-controlled sterile aDNA work space, wearing of protective clothing, using disposable tools, pipettes with aerosol resistant filter tips and treating equipment and working surfaces with bleach and ultra-violet irradiation frequently.

To ensure the authenticity of the mtDNA and Y-chromosomal sequences, and to detect possible PCR errors, each DNA fragment of each sample was sequenced from at least two different PCR reactions with DNA derived from different extractions. The sample was considered to be reproducible when consistent sequences of each DNA fragment were obtained from at least three amplifications. The consistent sequences were verified from two extractions in analyses done at least in two independent aDNA laboratories. Overlapping primers specific to cattle DNA were designed to prevent cross reactivity with human DNA (Text A in S1 File, Table B in S1 File). Negative controls were applied for all steps in the aDNA extraction and amplification. A previously analysed mammoth sample [31] was used as a positive control when the first five samples were extracted. The mammoth sample was suitable as a positive control as it is ancient and its sequence clearly differs from cattle.

For further analyses, sequences from aDNA samples obtained from different extractions and amplifications, proven identical by at least two independent aDNA laboratories were used. One sample was not repeatable and was thus excluded from analyses (Table A in S1 File). For six samples only partial mtDNA was successfully amplified. Consequently, they were omitted from the statistical analyses. As amplification from 25 samples (including one modern sample, Table A in S1 File) yielded no DNA, a total of 45 samples remained for statistical analyses (Table A in S1 File).

### Statistical analysis

The mtDNA sequences from the 45 successfully sequenced ancient cattle were aligned separately for the 486 bp D-loop and the 181 bp ND5 gene sequences using CLUSTALW [32] where penalties used were 10 for gap opening, 0.20 for gap extension, and 5 for gap distances. The combination of the sequenced regions is referred to below as the 667 bp haplotype region. A CLUSTALW alignment was also performed for the seven successfully amplified Y-chromosomal 155 bp sequences. The analysed sequences are available in GenBank, accession numbers KF233429-KF233528.

The Reduced Median-joining Network (RMN to be most conservative ε = 0) was constructed according to the algorithm described by Bandelt, Forster and Rohl [33] with NETWORK 4.6.0.0 [33]. The topology obtained in RMN was confirmed with the Maximum likelihood (ML) and Bayesian Markov Chain Monte Carlo (MCMC) analyses using jModeltest v2.1 [34], PhyML 3.0 [35] and MrBayes 3.2 [36]. Both the ML and the MCMC tree along with the detailed statistical methods are presented in Supporting Information (Text A in S1 File, Fig B in S1 File).

DnaSP (version 5) [37] was used to calculate the genetic diversity estimates based on the 486 bp D-loop sequences. Number of haplotypes (h), haplotypic diversity (Hd), number of segregating sites (S), nucleotide diversity (π), Tajima’s D (D), and average number of nucleotide differences (K) were calculated for each population. To approximate the level of bias in the diversity estimates caused by heterochronity in the dataset when pooling samples of different ages, corrected π_hμ_ [38] was calculated with mutation rates of 34 and 53% per million years and generation lengths of 5 and 7 years (upper and lower ranges as calculated from Near-Eastern cattle in [39]). In order to provide dates to the samples when calculating π_hμ_, radiocarbon dates were used and the used dates were randomly assigned to cover the range of context for samples dated by context.

In order to compare ancient cattle diversity to modern cattle populations, a number of additional sequences from Europe, Near East and North Asia were included in the population diversity analysis. These sequences have previously been described and analysed [10,11,15,16,20]. The size of the common aligned mtDNA sequence in this comparison was 245 bp from a total of 2139 individuals. This dataset was then used in two approaches.

First, to explore the temporal fluctuation in haplotypes within the N-EBSR, 49 modern cattle samples from five native N-EBSR breeds (Northern, Western, and Eastern Finncattle, Estonian Red and Estonian Native [11,16]), along with the 45 ancient cattle analysed here were extracted from the aligned 245 bp dataset. These 94 N-EBSR samples were grouped into three temporal cohorts; Prehistoric and Medieval (n = 19), Post-Medieval (n = 26), and Modern (n = 49) and into two groups: 1) the most frequent 245 bp haplotype found among the entire 2139 dataset (563 samples) and 2) the rest of the haplotypes.

The second approach was used to explore the appearance and frequency of ancient haplotypes among 2094 modern cattle divided into ten geographical regions (N-EBSR, Scandinavia, Western Europe, Southern Europe, South-Eastern Europe, Eastern Europe, Western Russia, Central Russia, Siberia, and Near East/Central Asia). For this approach, the 2094 modern samples were grouped into three haplotype groups: 1) the most common 245 bp haplotype in the entire dataset (563 out of 2139 samples), 2) the rest of the haplotypes found among 45 ancient N-EBSR cattle, and 3) other haplotypes not found in ancient data. The procedure was used to study the distribution of ancient haplotypes among contemporary cattle. Note that the Prehistoric haplotypes were excluded here as most of the Prehistoric haplotypes were not present in contemporary data.

Pearson’s chi-square test, as implemented in SPSS v.11.5.0, was conducted to test for differences in frequencies of mtDNA haplotypes in both approaches, between the temporal cohorts and the geographical regions.

Sixty-nine samples were further analysed for the Y chromosomal SNP in *UTY19*, which differentiates cattle Y chromosomes into haplotypes Y1 and Y2 [22]. The seven samples successfully analysed for the Y1/Y2 marker were analysed for temporal fluctuation with the Swedish ancient (n = 64) and Fennoscandian modern (Northern, Western, and Eastern Finncattle, Swedish Red, Red polled, Fjallnara and Mountain cattle, n = 41) data given in [22–25]. The combined data from Fennoscandian bulls were divided into four temporal groups: Iron Age (n = 8 [25]), Medieval (n = 37 this study and [24,25]), Post-Medieval (n = 19 this study and [24]), and modern (n = 28 [22] and [11] as reported in [23]). To compare the temporal analyses in Fennoscandia to Central Europe, data from Medieval bulls (n = 14, [26]) from Switzerland was analysed together with data from modern Swiss breeds (Braunvieh, Ehringer, and Simmental, n = 39, [22] and [11,40] as reported in [23]).

In order to make wider geographical comparisons, Y1/Y2 information from 127 modern Eurasian breeds (n = 1614 [22,24] and [23] combining the data of [11,40–42]) were included. Data from a total of 1692 bulls was divided into nine geographical regions (the Nordic countries, Western Europe, Southern and Central Europe, South Eastern Europe, Eastern Europe, Near-East and Central Asia, Western Russia, Central Russia and Siberia).

A Pearson’s chi-square test, as implemented in SPSS v.11.5.0, was conducted to test for differences in frequencies of Y1 and Y2 between the temporal cohorts (Fennoscandia and Switzerland) and geographical regions. In cases where 20% or more of the groups had expected counts less than 5, Fisher’s exact probability two-tailed test was used instead.

## Results

### Radiocarbon dating

A total of 21 samples were radiocarbon dated. Three samples appeared to be from a later period than expected based on the context dating while one sample from an Iron Age context turned out to be modern (Table A in S1 File).

### Osteological analysis

The metrical analysis of metacarpals revealed three males and 12 females while two metacarpals were indeterminable and five metacarpals were too fragmented to be analysed by osteological methods (Table A in S1 File). The results from the Y-chromosomal UTY19 were in accordance with the osteological analyses as none of the samples taken from female metacarpals amplified with Y-chromosomal primers. Two male metacarpals were confirmed and one indeterminable metacarpal was determined as male by Y-chromosomal amplification (Table A in S1 File).

### MtDNA haplotypes

**Fig 1:**
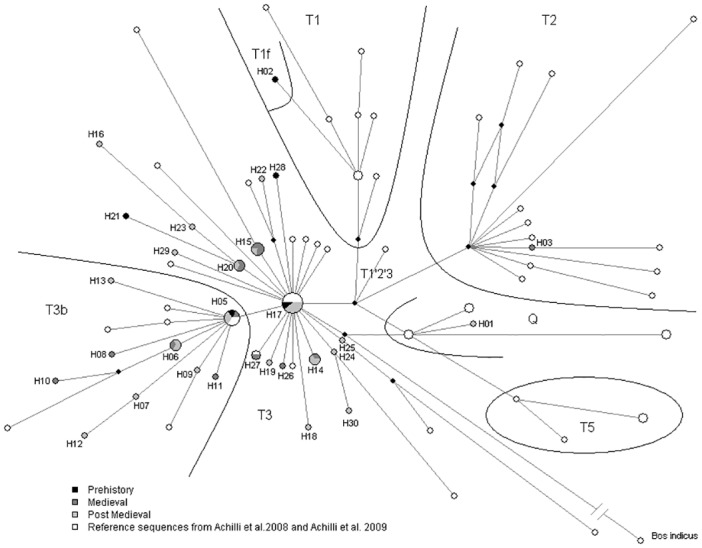
Median-joining network of the 29 ancient mitochondrial haplotypes (grey-black) with 43 modern reference haplotypes (white). Median-joining network (ε = 0) shows molecular relationships between 30 ancient haplotypes (H01-H03 and H05-H30). Major haplogroups (T1, T2, T3, T5 and Q) and sub-haplogroups (T1f, T3b) are defined by inclusion of 43 modern reference haplotypes from [10,15]. Each circle represents one mtDNA haplotype where the size is proportional to the number of individuals in that haplotype. Black diamonds represent hypothetical haplotypes. The length of the branches is proportional to the number of mutations between the haplotypes except the branch between *Bos taurus* and *Bos indicus* (32 mutations), which is shortened to fit in the picture. Haplotypes from the Prehistoric, Medieval, and Post-Medieval periods are indicated in black, dark grey, and light grey, respectively.

Using DnaSP, 30 haplotypes were found among the ancient cattle, including one sample providing only partial information. Twenty-nine haplotypes, including the full 667 bp sequence, were used for further analysis. When analysing the phylogeny of these 29 haplotypes, Bayesian MCMC, ML and RMN analyses gave similar topologies (Fig 1, Median-joining network of the 29 ancient mitochondrial haplotypes (grey-black) with 43 modern reference haplotypes (white), and Fig B in S1 File). All of the ancient haplotypes were assigned to the taurine haplogroups according to the known diagnostic positions of cattle mtDNA [10,11,14,15,20] (Text A in S1 File). One sample was assigned to taurine macro-haplogroup Q while the rest of the samples were assigned to the taurine macro-haplogroup T (Fig 1, Text A in S1 File). The 28 ancient haplotypes in macro-haplogroup T were further divided into haplogroups T2 (one haplotype) and T3 (17 haplotypes) and sub-haplogroups T3b (9 haplotypes) and T1f (one haplotype) (Fig 1, B and C Figs in S1 File, Text A in S1 File). The sample providing partial information was assigned to T2 (Text A in S1 File, Fig C in S1 File).

### Analysis of population diversity

The mtDNA diversity in the Finnish, Estonian, and Vyborg ancient cattle data are summarized in Table 1. The nucleotide diversity for the entire data set was 0.969. Within each ancient temporal cattle cohort the mitochondrial haplotype diversity estimates (s, h, Hd, K, and π) indicate a high diversity (Table 1). The haplotype diversity was highest in Prehistoric cattle (Hd = 1.000) and slightly lower in Medieval and Post-Medieval cattle (Hd = 0.956 and 0.972, respectively). Nucleotide diversity varied among periods with the highest observed diversity (π = 7.41* 10^–3^) in the Prehistoric population (Table 1). The bias in nucleotide-diversity estimate caused by heterochronity was low, less than 1.5% in all temporal cohorts (Table 1). Tajima’s D value was negative for all temporal cohorts with a significantly negative p value for the Post-Medieval period and the whole ancient cattle dataset suggesting a population expansion in Finland including Vyborg and the Baltic region (Table 1).

**Table 1:** Summary statistics of mtDNA variation in ancient North East Baltic Sea region cattle from Prehistoric, Medieval, and Post-Medieval periods.

	Ancient North East Baltic Sea region cattle			
	Prehistory, 700 BC-1200 AD	Medieval, 1200–1550 AD	Post-Medieval, 1200–1800 AD	Total
**N**	5	14	26	45
**S**	9	15	22	33
**h**	5	11	20	29
**Hd**	1.000	0.956	0.972	0.969
**K**	3.600	3.055	2.788	2.951
**θs**	4.320	4.717	5.765	7.736
**D**	-1.184	-1.437	-1.869*	-2.067*
**π**	7.41	6.29	5.74	6.07
**π**_**hμ**_ ^a^	7.35	6.28	5.73	6.04
**Bias** ^a^	0.84%	0.13%	0.10%	0.43%
**π**_**hμ**_ ^b^	7.30	6.28	5.73	6.03
**Bias** ^b^	1.45%	0.22%	0.17%	0.74%

N is number of individuals sampled; S is the number of segregating sites (excluding indels); h is the number of haplotypes; Hd is the haplotype diversity; K is the average number of differences; θs is ‘Theta’ derived from the observed number of segregating sites (*S*); D is Tajima′s D statistic value where statistical significances P<0.05 is marked with *.π is the nucleotide diversity*10^–3^; The Prehistoric cohort includes two samples from Late Bronze Age and three samples from Late Iron Age.

^**a**^Based on generation length of 7 years and mutation rate of 43% per million years

^**b**^Based on generation length of 5 years and mutation rate of 53% per million years

Haplogroup T3 and sub-haplogroup T3b formed a star-like phylogeny of haplotypes, with major haplotypes H17 and H05 for T3 and T3b, respectively. The highest haplotype diversity was detected in the oldest and smallest sample, from the Prehistoric period. A different set of haplotypes was found from the Medieval and Post-Medieval samples (Fig 1).

### Temporal mtDNA analyses

**Fig 2:**
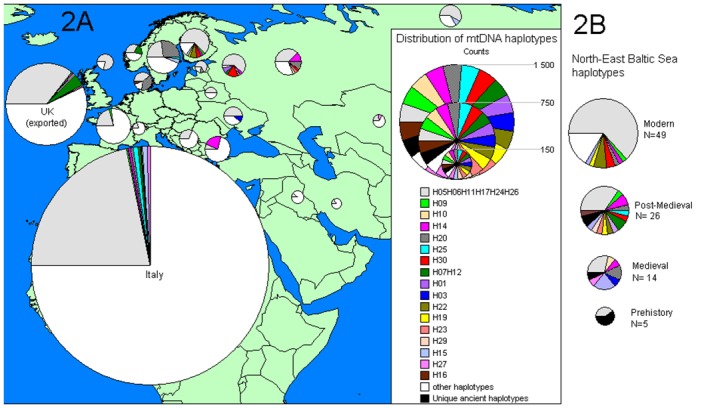
Distribution of ancient N-EBSR cattle mtDNA haplotypes in modern Eurasian cattle populations. Haplotype distribution in ancient Finnish, Estonian and Western Russian (Vyborg at the shore of Baltic Sea) cattle populations from the Late Bronze Age, Iron Age, Medieval, and Post-Medieval periods is indicated with pie charts at the right side of the map (2B, see Table C in S1 File). Seventeen ancient haplotypes found in modern Eurasian populations (Table C in S1 File) are indicated by pie charts with corresponding patterns (see key) on the map (2A). The modern haplotypes not found in ancient cattle are counted together and indicated in white. Counts of unique ancient haplotypes not found in modern populations are indicated in black.

Significant temporal fluctuations in the frequency of mtDNA haplotypes in the N-EBSR cattle were detected (Pearson Chi-Square test, n = 94, χ^2^ = 13.1, df = 4, p = 0.011). Here the most common 245 bp haplotype increased in frequency more than twice from Medieval to Post-Medieval and more than thrice from Post-Medieval to modern time (grey in Fig 2b in Fig 2, Distribution of ancient N-EBSR cattle mtDNA haplotypes in modern Eurasian cattle populations). Consequently, the proportion of other haplotypes decreased through time (coloured and white patterns in Fig 2b). Nearly half of these other haplotypes in contemporary cattle were not found in ancient cohorts (white in Fig 2b); and thus the proportion of the ancient haplotypes (other than the most common) in modern N-EBSR is approximately 20% (colored patterns in Fig 2b). The proportion of unique ancient haplotypes (unique among 2139 samples) was highest in the Prehistoric sample (black in Fig 2b).

### Geographical mtDNA analyses

The most common 245 bp haplotype (including the ancient 667 bp haplotypes H05, H06, H11, H17, H24, and H26, Table C in S1 File) was found in most modern European and Russian breeds with a frequency ranging from 16 to 63% within geographical regions (Table 2). The other ancient haplotypes had more restricted occurrences and frequencies, less than 1.6% among the 2094 modern cattle dataset (Table C in S1 File).

**Table 2:** Distribution of N-EBSR ancient haplotypes in modern European and Asian cattle breeds.

N-EBSR		Scandinavia	Western Europe	Southern Europe	South-Eastern Europe	Eastern Europe	Western Russia	Near East and Central Asia	Central Russia	Siberia	Total
Common H	31	23	93	334	8	13	16	4	12	14	548
	63.3%	28.4%	38.1%	21.7%	16.0%	50.0%	61.5%	16.0%	37.5%	58.3%	26.2%
Other Ancient H	10	27	17	51	7	2	9	1	10	2	136
	20.4%	33.3%	7.0%	3.3%	14.0%	7.7%	34.6%	4.0%	31.3%	8.3%	6.5%
H not found in Ancient data	8	31	134	1152	35	11	1	20	10	8	1410
	16.3%	38.3%	54.9%	75.0%	70.0%	42.3%	3.8%	80.0%	31.3%	33.3%	67.3%
**Total**	49	81	244	1537	50	26	26	25	32	24	2094

Figures represent the count and percentage of modern cattle data from ten geographical regions grouped in three haplotype (H) groups according to the appearance of the haplotypes in ancient N-EBSR data: The most common 245 bp haplotype (Common H), other ancient haplotypes found in Post-Medieval or Medieval periods and haplotypes not found (H not found) in ancient North-East Baltic Sea region cattle.

There were significant differences in appearance and frequency of ancient haplotypes among ten geographical regions of contemporary cattle (Pearson Chi-Square test, n = 2094, χ^2^ = 355, df = 18, p<0.001). The proportion of ancient haplotypes was highest in contemporary N-EBSR cattle and Western Russian cattle (Table 2), while the proportion of haplotypes not found in our ancient sample increased with geographical distance showing highest proportions in South and South-East Europe, and Near East/Central Asia (Table 2, indicated in white in Fig 2a).

### Y-chromosomal analysis

UTY19 allele frequencies in Fennoscandian cattle (Table D in S1 File) differed significantly between temporal cohorts (Chi-Square test, p<0.001). Type Y2 was dominating in both the Iron Age (7/8) and the Medieval period (36/37), with no statistical difference in allele frequencies between the two periods (Fisher’s Exact test, p = 0.327). The proportion of Y1 increased significantly from the Medieval (1/37) to the Post-Medieval period (9/19, Fisher’s Exact Test, p<0.001) and then again from the Post-Medieval period (9/19) to Modern times (33/41, Fisher’s Exact Test p = 0.015). The Y1 type was fixed in most contemporary Fennoscandian native breeds with only one exception where Y2 was dominating (8/9), viz. in one Finnish breed, the Eastern Finncattle.

There was no significant temporal changes detected in Central Europe (Switzerland) from Medieval (late 13^th^ century) to modern times (Fisher’s Exact Test, p = 0.462), where Y2 dominated both the Medieval (13/14) and the modern (38/39) periods.

Y1 and Y2 haplotype frequencies varied significantly between geographical regions (Chi-Square test, p<0.001, Table 3, Table E in S1 File). Most of the modern breeds (105 from 127) in all regions were fixed for one Y-haplotype, either Y1 (46) or Y2 (59), while 22 displayed both Y1 and Y2 (Table E in S1 File).

**Table 3:** Summary of ancient and modern Y-haplotypes distribution across Eurasia.

	Nordic counties	Western Europe	Southern and Central Europe	South-Eastern Europe	Eastern Europe	Western Russia	Near East and Central Asia	Central Russia	Siberia	Total
Ancient										
Y1	11		1							
	17%		7%							
Y2	53		13							
	83%		93%							
Total	64		14							78
Modern										
Y1	101	334	120		53	9	1	24		
	84%	83%	13%		82%	100%	3%	96%		
Y2	19	70	806	10	12		31	1	23	
	16%	17%	87%	100%	18%		97%	4%	100%	
Total	120	404	926	10	65	9	32	25	23	1614

Data includes 78 ancient (from Finland, Sweden and Switzerland) and 1621 modern Eurasian bulls. Separate figures for each breed and ancient populations are given in Table E in S1 File.

## Discussion

### MtDNA haplogroups

The assignment of ancient samples into bovine haplogroups (Q, T2, T3) or sub-haplogroups (T1f, T3b), with T3 and T3b predominating, is in good agreement with population analysis of modern cattle, where T3 is the major mtDNA haplogroup in Eurasian populations [11,14]. It is also in accordance with previous analysis of ancient European cattle populations where a predominance of the T3 haplogroup has been shown from the Neolithic [19].

A rare haplotype, belonging to sub-haplogroup T1f, was found in a sample dated to the Late Bronze Age in Estonia at a frequency of 1/5 in the Prehistoric cohort (Fig 2). In a previous study, T1f has been found in three individuals from the modern Italian breed Podolian (3/80 of T1 haplogroup sequences found in Europe) and in the modern breed Menofi from Egypt (frequency 1/196 of T1 haplogroup sequences found in Africa) [20]. Taking into account that Bonfiglio *et al*. [20] analysed more than two thousand mtDNA samples in order to obtain 54 T1 haplotypes, the frequency of T1f must be less than 4/2000 among European, African, and American cattle breeds.

Haplogroups Q and T2 were rare in the ancient cattle populations in the N-EBSR just as they are in contemporary populations [11]. Haplogroup Q was found in the Northern Finnish Post-Medieval population at a frequency of 1/26; it has previously been found in five Italian native cattle breeds [10,15,16]. Most previous studies, however, failed to differentiate haplogroup Q from haplogroup T, as they overlooked the sequence of the diagnostic site outside the D-loop [2,11,19]. Based on other D-loop defining positions typical to haplogroup Q, the distribution of haplogroup Q is suggested to cover at least several South European countries, Egypt, Turkey, and China [16].

Mitochondrial haplogroup T2 has been found in France from bones dating to 5000 BP [43] and in bones from Switzerland dating to the Roman period [44], as well as in contemporary Swiss cattle in two haplotypes derived from the central T2. Here the authors [44] concluded that the Near eastern T2 lineage was introduced to Switzerland during Roman times or earlier.

According to our knowledge, this is the first time that haplogroup Q and T1 have been found in any ancient or modern Northern European cattle population. The existence of these rare haplotypes, as well as the T2-related haplotypes in the ancient data suggests that the ancient cattle population in the N-EBSR was different from modern cattle.

### Y—chromosome

The significant increase of Y1 and decrease of Y2 in Finnish bulls from the Post-Medieval period to the present is in accordance with a similar temporal shift of paternal lines detected in Swedish cattle [24]. The higher proportion of haplotype Y1 in Swedish Post-Medieval bulls compared to Finnish bulls from the same time period may be due to the small sample size of Finnish ancient bulls, or it may indicate that the replacement of Y2 with Y1 happened later in Finland than in Sweden. Multiple arrivals of cattle to the Nordic regions have been suggested, although the timing of the arrival of Y1 to the Nordic regions could not be determined based on modern samples [23]. This study, in accordance with previous studies [24,25], suggests that this replacement of Y2 with Y1, resulting in an almost complete fixation of the Y1 type in the contemporary Fennoscandia [22,23], goes back 600 years starting at the turn of the Medieval and the Post-Medieval periods with an accelerating speed during the past 200 years. This is also in accordance with written historical records [45]. Import of foreign cattle from countries such as the Netherlands and Sweden, especially bulls, increased in Finland during the 18^th^— 19^th^ centuries [45]. In contemporary Taurus cattle populations, haplotype Y2 dominates in almost the whole Eurasian region [23,46,47], with the exception of Western and Northern Europe [23]. The present-day European Y-chromosomal distribution has been taken as evidence of two different expansions of dairy cattle to the Nordic countries [23]. Results from this study suggest that the replacement of Y1 haplotypes in Fennoscandia is a quite recent phenomenon.

### Temporal fluctuation in the N-EBSR cattle

After considering the possible reasons for the observed variations in haplotype diversity and frequencies (sampling effect, contamination, deamination, mutations, selection, and migration, see supporting discussion in Text A in S1 File), the most plausible explanations for the observed temporal changes in this data is migration and selection of breeding animals.

Our data suggests a temporal fluctuation in cattle populations in the northern Baltic Sea region. The small set of Prehistoric samples displayed different haplotypes (Hd = 1) with an equal amount of haplogroups and sub-haplogroups (three haplo-/ sub-haplogroups, T3, T3b and T1f, Fig 1) compared to larger samples of later periods. The Finnish Prehistoric samples were from the Late Iron Age (800–1200 AD), during which the human population of the Western Finnish Iron Age culture increased and also dispersed further east and north [48].

A different set of haplotypes were detected in the N-EBSR from the Medieval and Post-Medieval samples. The proportion of mtDNA haplotypes also found in modern Eurasian cattle populations was higher, although there was a small amount of unique ancient haplotypes (Fig 2a and 2b, Table 2). Three haplogroups and sub-haplogroups were present in the Medieval (T3, T3b and T2) and Post-Medieval (T3, T3b and Q) periods. Haplogroup Q and the phylogenetically oldest T3 haplotypes may originate from early Southern European populations where they are still found at low frequencies. In addition, the rare Post-Medieval T3-haplotypes, H22, H30, and H25 have counterparts (based on 206 bp and 146 bp common sequences) in Neolithic and Bronze Age German samples [19] and in samples from Early Medieval Scandinavian settlements in Dublin, Ireland [2], supporting the idea of an European origin of the Medieval and Post-Medieval N-EBSR cattle. The few contemporary rare T2 haplotypes from Switzerland have been hypothesized to date back to the introduction of T2 into Central Europe before or during Roman times [44]. Simultaneously, the frequency of the Y-chromosomal haplotype Y1, common in modern Northern European cattle [22,23], increased in northern Baltic Sea region cattle (Table D in S1 File).

Detected temporal changes in mtDNA and Y-chromosomal haplotypes are in accordance with historical events. During the Medieval period there were major changes in the society in N-EBSR. The population density and the intensity of cultivation increased and settlements spread to previously uninhabited areas in between old settlement cores [49,50]. Cultural connections and migration routes at N-EBSR changed due to the reign of the Swedish Kingdom, and in Estonia by reign of multiple forces e.g. the Teutonic Order [51]. The old Iron Age culture was suppressed, a church organisation was established and towns were founded [51]. The new cattle haplotypes found in the N-EBSR medieval samples could thus relate to migration from Southern and Western Europe, especially from Sweden and Germany, but during the 12^th^ and 13^th^ centuries possibly also from Russia, to Finland and Estonia [51,52].

After the war between Sweden and Russia in 1721, there was an increase in influence from the east on the N-EBSR. This is when the Baltic countries and the South-East part of Finland came under Russian rule, just like the rest of Finland at the end of the Post-Medieval period in 1809. The most severe starvation period in North European history (1695–1697 AD) [53] predated the change in reign in 1721, thus giving room for replacement with new cattle material.

The latest shift in mtDNA haplotypes, viz. from the Post-Medieval period to the present can be seen in Fig 2. The resemblance of mtDNA haplotypes and their frequencies between modern Finnish native breeds and modern Western Russian breeds (previously reported in [11]) can be seen in Fig 2a. However, the ancient N-EBSR population resembles the modern West Russian population more closely than the modern N-EBSR cattle populations (Table 2). This fits well with the historical written sources, since only some cattle were imported from Russia during the 19^th^ century, when most of the new stock originated from the Netherlands, British Isles, Germany and Sweden [45]. In a recent autosomal SNP marker study, a western European origin is also suggested for two modern Baltic breeds, Lithuanian Light Grey and Lithuanian White Backed [54]. The latest shift from the Post-Medieval to the present can also be explained by a growing interest in specialised breeds during the 19^th^ and 20^th^ century and by more efficient breeding methods leading to stronger selection.

## Conclusions

Analyses of ancient cattle remains in this study revealed important trends in the history of the N-EBSR cattle: 1) The rare haplogroup Q was detected in Northern Europe for the first time, indicating that besides the taurine T-haplogroups, haplogroup Q also reached the peripheral area of the Baltic Sea region no later than in the Post-Medieval period (1550–1800 AD). The rare Q and T1f haplotypes found in ancient cattle have not been detected in the Scandinavian region in contemporary cattle nor in Middle European populations. 2) Genetic diversity has decreased over time from Prehistory up until modern times. The observed replacement of Prehistoric cattle haplotypes with modern haplotypes may result from population bottlenecks caused by demographical events in the Medieval and Post-medieval periods and/or changes in cultural connections and thus migration of cattle in the N-EBSR. However, the recent loss of genetic variation may be due to an increased interest in and selection for specialized breeds. 3) Modern and ancient counterparts of mtDNA haplotypes suggest a European origin for Medieval N-EBSR cattle, with increasing Eastern influence during the Post-Medieval period. The small sample of prehistoric N-EBSR cattle displayed both the most widespread T3 haplotype and haplotypes not yet found in previous studies. Thus the origin of Prehistoric N-EBSR cattle remains uncertain. Future ancient and modern mtDNA studies detecting the rest of the Prehistoric haplotypes may shed more light on the origins of prehistoric N-EBSR cattle.

## Supporting Information

S1 Dataset Alignments of 965 mtDNA and Y-chromosomal amplicons from 45 ancient samples included in the statistical analyses. (TXT)

S1 File Supporting Information includes Supporting Text (Text A), three Supporting Figures (A-C Figs) and five Supporting Tables (A-E Tables). Supporting text provides information about primer design, supporting statistical methods, and supporting discussion (Text A). Supporting figures gives locations of excavation sites (Fig A), phylogeny of haplotypes (Fig B), and alignment of sequences (Fig C). Supporting tables summarises detailed information of each ancient sample (Table A), and primers (Table B) used in the present study. Datasets used in the statistical analyses are provided in C-E Tables in S1 File.
(PDF)
