# Supplementary material for: Temporal Fluctuation in North East Baltic Sea Region Cattle Population Revealed by Mitochondrial and Y-Chromosomal DNA Analyses
Source: PLoS One. 2015 May 20;10(5):e0123821. doi: 10.1371/journal.pone.0123821 (PMC4439080; doi:10.1371/journal.pone.0123821)
Supplement: S1 File — Supporting text provides information about primer design, supporting statistical methods, and supporting discussion (Text A). Supporting figures gives locations of excavation sites (Fig A), phylogeny of haplotypes (Fig B), and alignment of sequences (Fig C). Supporting tables summarises detailed information of each ancient sample (Table A), and primers (Table B) used in the present study. Datasets used in the statistical analyses are provided in C-E Tables in S1 File. (PDF) [file pone.0123821.s002.pdf]

SUPPORTING INFORMATION, S1 FILE

Text A. Supporting methods and discussion.

Supporting text provides detailed information about primer design, supporting statistical methods and discussion about sampling effect, contamination, deamination, mutations, and migration.

Primer design

Primers (see Table B in S1 File) used in the study were designed with Primer3 (http://primer3.sourceforge.net/) using mtDNA [GenBank: V00654] and UTY19 [GenBank: AY936543] gene reference sequences.

Success rate of aDNA analyses

From a total of 77 ancient cattle samples subjected to mtDNA analysis, mtDNA amplification succeeded in 51 samples, of which six yielded only partial mtDNA sequences (Table A in S1 File). Table B in S1 File summarises the average amplification success and fragment lengths for the cattle mtDNA sequences. Forty-five mtDNA samples remained for statistical analyses, five from the Prehistoric, 14 from Medieval, and 26 from Post-Medieval period. The 155-bp sequence of intron 19 in the *UTY* gene was analysed in 67 samples and seven of them yielded the *UTY19* intron sequence. The lower success rate detected in the amplification of Y-chromosomal marker was expected as Y-chromosome can only be found in males and nuclear DNA is harder to retrieve from ancient samples than mtDNA.

Definition of ancient haplogroups and sub-haplogroups

Bayesian MCMC, ML, and RMN analyses confirmed the assignment of 29 ancient haplotypes into taurine haplogroups (Fig. B in S1 File, Fig.1 in main text). To divide haplogroups further into known sub-haplogroups, we studied nucleotide differences at the defining diagnostic positions [1-4]. In total nine haplotypes were assigned to the T3b sub-group and 17 to the T3 (defining position 169 in V00654, Fig. C in S1 File). One sample was assigned to haplogroup T2 (defining positions 16057 [G>C transversion], 16185, and 16255) (Fig. C in S1 File). Two of these T2 diagnostic mutations (transitions at positions 16185 and 16255) defined one sample with partial information to haplogroup T2 (Fig. C in S1 File). One sample (H01 in Fig. C in S1 File) was assigned to belong to either haplogroup T or Q (defining nucleotide positions 16255 and 13005). Transversion C>G at position (15953) in the additional 77 bp D-loop fragment (positions 15936-16012 in V00654) confirmed the sample to belong to haplogroup Q (Fig. C in S1 File).

A Prehistoric Estonian sample assigned to haplotype H02 could not be ascribed to any of the known T subclades above. Diagnostic markers (defining positions 169, 16255, 16050, and 16113 in V00654) within the D-loop indicated that H02 belongs to the recently found sub-haplogroup T1f, having the same reversion at the diagnostic site (@16113) according to Bonfiglio et al. [5]. Within the region analysed here (486 + 181 bp) H02 differs by one mutation (a transition at position 16302) from two known sub-haplogroup T1f sequences [JN817329.1] and [JN817343.1] [5].

Additional methods; construction of phylogenetic three

The most appropriate model of DNA substitution among 88 candidate models on a Maximum

Likelihood (ML) tree under the Bayesian Information Criterion (BIC), Akaike Information Criterion (AIC) and corrected AIC(c) was determinate with jModeltest v2.1 [6]. Hasegawa-Kishino-Yano model with invariant sites (I) and gamma distributed rates (G) (HKY+I+G) with a gamma value of 0.6420 was the best fit model according to BIC and was also suggested as a second best model by AIC and AIC(c). According to AIC alone, the best-fit model of DNA sequence evolution was a Kimura 3-parameter model with unequal base frequencies (uf), invariant sites, and gamma distributed rates (TPM1uf+I+G) with a gamma value of 0.6800.

Genetic distances for the 45 ancient cattle mtDNA sequences were estimated using ML analysis with a HKY+G+I substitution model and a gamma parameter of 0.6420. To generate an initial tree for the ML analysis, a model averaged phylogeny was calculated in jModelTest v2.1 from 88 candidate models using BIC as a selection criterion, a confidence interval of 1.00, and with a 50% majority rule in the 1.00 confidence interval. ML Bootstrap values were calculated with 1000 replicates using PhyML 3.0 [7]. The tree was drawn using FigTree 1.3.1. (http://tree.bio.ed.ac.uk/software/figtree/). The sequence of *Bos indicus* [FJ971088] was used as an out-group in the phylogenetic analysis of haplotypes. Reference sequences for taurine haplogroups T1 [EU177841, EU177843-EU177848], T2 [EU177849-EU177859, EU177861], T3 [EU177816-EU177820, EU177824-EU177830, EU177832, EU177836 -EU177839], T1'2'3 [EU177840], T5 [EU177863 -EU177865], Q [FJ971081, FJ971082, EU177866], and R [FJ971086-FJ971087] were also included.

The ML tree topology was confirmed by Bayesian Markov Chain Monte Carlo (MCMC) analyses using MrBayes 3.2 [8]. Bayesian MCMC analyses were conducted using theHKY+G+I model. Three million generations in four independent MCMC analyses were conducted with sampling every 100 generations and the first 25 % was discarded as burn-in. When the potential scale reduction factor values approached 1.0 and the average standard deviation of split frequencies fell close to 0.01, Markov chain stationary was considered to be reached.

Post-Mortem degradation and contamination

The post mortem degradation of DNA by endogenous nucleases as well as physical oxidative and hydrolytic damages may cause errors in PCR especially in very old specimens (tens of thousands years) [9]. Samples included in the current study derived mostly from the last few hundred years with the oldest samples from the Late Bronze Age (800-600 BC). All the ancient samples included in the statistical analyses were repeatable with no signs of deamination or contamination. The ancient cattle sequences showed reasonable molecular behaviour as they were assigned to previously found taurine T and Q mega-haplogroups with most of the haplotypes having counterparts in modern cattle (discussed in the main text). In addition, post-mortem degradation of DNA should affect nucleotides and sugar-phosphate back bone of the DNA strand [9] over the whole length of the strand, not only at the diagnostic motifs. Therefore it is unlikely that haplotypes detected in the current study would be a result of post mortem degradation.

Sampling effect, newborn haplotypes, selection or migration?

Major concern was taken to take representative sample from ancient N-EBSR cattle and minimize the possibility to sample close relatives (Table A in S1 File). Ancient samples from all excavation sites where more than one individual was analysed showed variation in mtDNA haplotypes. The ancient bulls in this study were from different excavation sites and time periods, and thus it is unlikely that they were close relatives. Two exceptions are a pair of Post-

Medieval bulls from Pietarsaari, Finland (BtPie1 and BtPie2, Table A in S1 File), but in this case both paternal lines Y1 and Y2 were detected.

We realize that the sampling effect and heterochronic nature of the data may affect the results. However, the time span of sample cohorts in this study was quite narrow: around 300 years for Medieval and Post Medieval periods, which corresponds to around 60 generations and 1900 years for the Prehistoric period. The longer time period in the Prehistoric cohort is due to the inclusion of two Bronze Age samples. As all the haplotypes in the Prehistoric cohort were different (Hd=1, Table 1 in main text) a summary of the genetic diversity gave similar statistical results for the total Prehistoric cohort as for the 300 years long Iron Age period. Moreover, the time period from the end of the Post-Medieval period to the present is around 200 years. Thus, when sampling the same N-EBSR, modern cohort corresponds to changes that occurred during the last 200 years. These time frames are too short for a major accumulation of new haplotypes by mutations as shown in the median joining network (Fig. 1 in main text), where the new haplotypes detected at later time periods do not form star-like patterns around any older haplotypes, as expected if they were produced by new mutations. In addition, the bias between uncorrected and heterochronity corrected nucleotide diversity estimates were marginal (Table 1 in main text).

A sampling effect may drop out rarer haplotypes in each temporal cohort, including modern samples, which may result in stochastic fluctuations in the distribution of haplotypes between temporal periods. The expected result of a sampling effect is therefore a decrease in the diversity of each sample especially in the smallest sample sets. In this data, a χ^2 -test was performed to statistically test the possibility that results were due to a sampling effect. Significant results rejected the null hypothesis of equal frequencies between periods, and in fact revealed an opposite pattern. Due to a temporal increase in the proportion of one haplotype, and a decrease in others (Fig. 2b in main text, see results), smaller data sets of ancient cohorts displayed higher diversity with more haplogroups observed compared to modern N-EBSR cattle population (see results in main text).

Selection of breeding animals or stochastic events in breeding populations may result to increase of certain haplotypes. In this data, artificial selection may have affected the haplotype diversity of the modern cohort, as the selection for specialised breeds started around 1900 when the herd books were established. Families with desired characters may be linked to certain haplotypes. In historical periods, selection of breeding animals may have happened more by change (i.e. genetic drift). For example, the most severe starvation in N-EBSR history in 1695-1697 AD likely caused a strong population bottleneck in all domestic animals. During these three exceptionally cold years, one third of the local human population died of starvation and a large proportion of the livestock was eaten [10]. After this kind of stock loss, any surviving animal was likely used for breeding. Whether the selection is intended or caused by drift, the signatures detected from population genetics would be similar, e.g. increase of the frequency of certain haplotype(s), as detected in this study (see the main text).

After considering the stochastic fluctuation due to a sampling effect and the possibility of new haplotypes evolving by mutations within the time frame of this data, the most plausible explanation for the observed temporal changes in this data is migration. The haplotypes detected in each period likely arrived to N-EBSR from other locations rather than evolving there. The temporal increase in frequency of certain haplotypes may be due to bottlenecks and/or artificial selection of breeding animals.

References in Supporting Information

1. Troy CS, MacHugh DE, Bailey JF, Magee DA, Loftus RT, Cunningham P, et al. Genetic evidence for Near-Eastern origins of European cattle. Nature. 2001;410: 1088-1091.
2. Achilli A, Olivieri A, Pellecchia M, Uboldi C, Colli L, Al-Zahery N, et al. Mitochondrial genomes of extinct aurochs survive in domestic cattle. Curr Biol. 2008;18: 157-158.
3. Achilli A, Bonfiglio S, Olivieri A, Malusa A, Pala M, Hooshiar Kashani B, et al. The multifaceted origin of taurine cattle reflected by the mitochondrial genome. PLoS One 2009 Jun 01. doi: 10.1371/journal.pone.0005753
4. Kantanen J, Edwards CJ, Bradley DG, Viinalass H, Thessler S, Ivanova Z, et al. Maternal and paternal genealogy of Eurasian taurine cattle (*Bos taurus*). Heredity. 2009;103: 404-415.
5. Bonfiglio S, Ginja C, De Gaetano A, Achilli A, Olivieri A, Colli L, et al. Origin and spread of *Bos taurus*: New clues from mitochondrial genomes belonging to haplogroup T1. PLoS One. 2012 Jun 07. doi: 10.1371/journal.pone.0038601
6. Posada D. jModelTest: Phylogenetic model averaging. Mol Biol Evol. 2008;25: 1253-1256.
7. Guindon S, Dufayard JF, Lefort V, Anisimova M, Hordijk W, Gascuel O. New algorithms and methods to estimate maximum-likelihood phylogenies: Assessing the performance of PhyML 3.0. Syst Biol. 2010;59: 307-321.
8. Ronquist F, Teslenko M, van der Mark P, Ayres DL, Darling A, Hohna S, et al. MrBayes 3.2: Efficient Bayesian phylogenetic inference and model choice across a large model space. Syst Biol. 2012;61: 539-542.
9. Hofreiter M, Serre D, Poinar HN, Kuch M, Paabo S. Ancient DNA. Nat Rev Genet. 2001;2: 353-359.
10. Muroma S. Suuret kuolonvuodet 1696-1697. In: Karonen P, editor. “Pane leipään puolet petäjäistä” – nälkä ja pulavuodet Suomen historiassa. University of Jyväskylä: Suomen historian julkaisuja 19; 1994. pp. 25-42.
11. Pihlman A. Åbo Akademin päärakennuksen tontin kaivaukset osana Turun kaupunkiarkeologiaa. In: Seppänen L, editor. Kaupunkia pintaa syvemmältä – Arkeologisia näkökulmia Turun historiaan. Turku: Archaeologia Medii Aevi Finlandiae 9; 2003. pp. 69-76.
12. Niukkanen M. Sirpaleita suurvalta-ajan Helsingistä. Hämeenlinna: Karisto Oy; 2002.
13. Vuorinen J. Rakennukset ja rakentajat Raision Ihalassa rautakauden lopulla ja varhaisella keskiajalla. Doctoral dissertations, University of Turku, Annales Universitatis Turkuensis C 281; 2009. Available: http://urn.fi/URN:ISBN:978-951-29-3900-8
14. Schultz EL. Mikkeli Moisio Latokallio / Moisionpelto ja Mikkelin Mlk Kyyhkylä / Porrassalmenpelto. Finnish National Board of Antiquities, Department of Archaeology; 1993.
15. Uotila K. Hautausmaan kaivaukset vuosina 2005-2007. In: Uotila K, editor. Naantalin luostarin rannassa. Stranden vid Nådendals kloster. Eura: Kåkenhus - kirjat nro 3; 2011. pp. 183-200.

16. Kallio T. Oulu, Oulun kadut: Oikokatu, Kajaaninkatu, Torikatu, Ojakatu, Saaristonkatu, Franzenin puisto. Katutöiden arkeologinen valvonta. Finnish National Board of Antiquites, Department of Monuments and Sites; 2005.

17. Kallio-Seppä T. Oulu, Lyseo 1/11/1. Kaupunkiarkeologinen koekaivaus 27.4.-21.5.2007. Finnish National Board of Antiquites, Department of Monuments and Sites; 2007.

18. Oikarinen T. Pietarsaari, 2/2/7-10, Lassfolkin kortteli (PSL-08). Kaupunkiarkeologinen pelastuskaivaus 19.5.-8.8.2008. Finnish National Board of Antiquites, Department of Monuments and Sites; 2009.

19. Poutiainen H. Sysmä Ihananiemi. Arkeologinen koekaivaus rautakautisella muinaisjäännösalueella. Lahti City Museum and Päijät-Häme Provincial Museum; 2000.

20. Herva VP. Kaupunkiarkeologinen pelastuskaivaus, Tornion Keskikatu 29-35. University of Oulu, Archaeological laboratory; 2002.

21. Alenius T, Laakso V. Palaeoecology and archaeology of the village of Uukuniemi, Eastern Finland. Acta Borealia. 2006;23: 145-165. doi: 10.1080/08003830601026834

22. Koivisto A. Haminan korttelin 23 koekaivaukset vuonna 2008. Finnish National Board of Antiquites, Department of Monuments and Sites; 2008.

23. Schultz E, Schultz H. Hämeenlinna Varikkoniemi – Eine späteisenzeitliche-frühmittelalterliche Kernsiedlung in Häme. Die Ausgrabungen 1986-1990. Suomen Museo: Suomen Muinaismuistoyhdistys; 1992;99: 41-85.

24. Gustavsson K. Franciskanerkloster på Kökar. Nytt ljus över medeltiden i Skärgårdshavet. Historisk Tidskrift för Finland. 1994;79: 494-518.

25. Pihlman A, Saloranta E, Ainasoja M, Hukantaival S, Lompolo V, Martiskainen H. Turku II, Pinella. Kaupunkiarkeologinen tutkimus 2010. Archives of Museum Center of Turku; 2010.

26. Hyttinen M. Tornio Suensaari YIT:N tontti (II/2/4). keskikatu 12. Kaupunkiarkeologiset koekaivaukset 24.5.-4.6.2010 ja kaivaukset 7.6.-6.7.2010. Finnish National Board of Antiquites, Department of Monuments and Sites; 2010.

27. Lang V. The bronze and early iron ages in Estonia. Estonian archaeology 3. Tartu: Tartu University Press; 2007.

28. Haak A. Archaeological investigations at Viljandi castle of the Teutonic Order and in medieval Viljandi. In: Tamla Ü, editor. Arheoloogilised välitööd Eestis = Archaeological Fieldwork in Estonia 2003. Tallinn: Muinsuskaitseamet; 2004. pp. 107-122.

29. Jaanits L, Laul S, Lõugas V, Tõnisson E. Eesti esiajalugu. Tallinn: Eesti Raamat; 1982.

30. Tamla T. Seliše Pada. Eesti Teaduste Akadeemia Toimetised. 1983;32: 302-306.

31. Tõnisson E. Eesti muinaslinnad. Muinasaja teadus. 2008;20: 266-269.

32. Valk H. Excavations in the ruins of Vastseliina Castle and on the hillforts of Urvaste and Hinniala. In: Tamla Ü, editor. Arheoloogilised Välitööd Eestis=Archeological fieldwork in Estonia 2001. Tallinn: Muinsuskaitseamet; 2007. pp. 49-67.

33. Saksa A, Belsky S, Kurbatov A, Polyakova N, Suhonen M. New archaeological excavations in Viipuri. Results of field investigations of the 1998 - 2001 seasons and current research problems of urban history. Fennoscandia Archaeologica. 2002;19: 3-30.

34. Lehtosalo-Hilander PL. Luistari/I. The Graves. Helsinki: Suomen muinaismuistoyhdistyksen aikakauskirja 82:1; 1982.
35. Herva V. Oulu Pikisaari, historiallisen ajan kaivaustutkimus 15.-26.5.2006. University of Oulu: Archaeological laboratory; 2006.
36. Miettinen M. Pihtiputaan Hämeensaari. Uutta tietoa Keski-Suomen historiasta. In: Purhonen P, editor. Lapinraunioita ja Hiidenkiukaita. Finnish National Board of Antiquites: Department of Monuments and Sites; 1993. pp. 52-64.
37. Ruohonen J. Hirvensalmi Vahvajärvi Lampuunlahti. Arkeologinen tarkastus 2010. Archives of Department of Archaeology, University of Turku; 2010.
38. Hautio M. Lieto Vanhalinna, Aittamäki. Kaivausraportti. Myöhäisrautakautisen polttokenttäkalmiston tasokaivaus. Archives of Archaeological department, University of Turku; 1994.
39. Antikainen A. Vesilahti, Hinsala, Tonttimäki. Rautakautisen kalmiston kaivaus 28.7.–31.8. 1986. Archives of Department of Archaeology, University of Turku; 1987.
40. Schultz EL. Helsinki Vartiokylä Linnavuori. Keskiaikaisen puolustusvarustuksen kaivaus. Archives of Helsinki City Museum; 2003.
41. Lahti E. Enontekiön Markkina. Markkinapaikan elämää arkeologisen tutkimuksen perusteella. MA Thesis, Department of Archaeology, University of Helsinki; 2004.
42. Leppäaho J. Mikkelinpitäjän Invaliidikoti Kyyhkylän (ent. Kyyhkylän kartano) Porrassalmen pellolla oleva rautakautinen kalmisto. Finnish National Board of Antiquites: Department of Monuments and Sites; 1939.
43. Uotila K. Turun Aboa Vetus -museon (Rettigin palatsin) kaivaukset vuosina 2009-2010. SKAS 2011. pp. 3-14.
44. Poutiainen H. Sulkava Iitlahden Hovi Keskipelto. Rautakautisen muinaisjäännöksen koekaivaus kesäkuussa 1992. Finnish National Board of Antiquites: Department of Monuments and Sites; 1993.
45. Kumpulainen M, Miettinen M. Pihtipudas Niemenharju 1 Niemi. Finnish National Board of Antiquites: Department of Monuments and Sites; 2008.
46. Luoto K. Sastamala Vehmaa (Vehmaa) kylätontin pelastuskaivaus 2009. Archives of Pirkanmaa Provincial Museum; 2010.
47. Peets J, Allmäe R, Maldre L. Archaeological investigations of Pre-Viking Age burial boat in Salme village at Saaremaa. Arheoloogilised välitööd Eestis = Archeological fieldwork in Estonia 2010 Tallinn: Muinsuskaitseinspektsioon; 2011: 29-48.
48. Valk H. Aruanne arheoloogilistest kaevamistest Viljandi Jaani kiriku kommunikatsioonidetrassil. Tallinn: Manuscript in the archaeology archive of Tartu University; 1991.
49. Valk H. Aruanne arheoloogilistest uurimistöödest Viljandis Suusahüppemäe piirkonnas 04.08.-06.09. Tallinn: Manuscript in the archaeology archive of Tartu University; 1999.
50. Vaba A, Valk H. Prehistoric settlements in Viljandi. New evidence. In: Tamla Ü, editor. Arheoloogilised välitööd Eestis=Archaeological field works in Estonia 2001. Tallinn: Muinsuskaitseinspektsioon; 2002. pp. 82-90.

51. Haak A, Valk H. Archaeological investigations of medieval and post-medieval Viljandi. In: Tamla Ü, editor. Arheoloogilised välitööd Eestis=Archaeological field works in Estonia 2001. Tallinn: Muinsuskaitseinspektsioon; 2002. pp. 91-104.

52. Valk H. Excavations in Viljandi: New data about the final period of Iron Age and the besieging of 1223. In: Tamla Ü, editor. Arheoloogilised välitööd Eestis=Archaeological field works in Estonia 2002. Tallinn: Muinsuskaitseamet; 2003. pp. 56-70.

53. Bonfiglio S, Achilli A, Olivieri A, Negrini R, Colli L, Liotta L, et al. The enigmatic origin of bovine mtDNA haplogroup R: Sporadic interbreeding or an independent event of *Bos primigenius* domestication in Italy? PLoS One 2010 Dec 28. doi: 10.1371/journal.pone.0015760

54. Telldahl Y, Svensson E, Götherström A, Storå J. Typing late prehistoric cows and bulls-osteology and genetics of cattle at the Eketorp Ringfort on the Öland island in Sweden. PLoS One 2011 Jun 22. doi: 10.1371/journal.pone.0020748

55. Svensson E, Götherström A. Temporal fluctuations of Y-chromosomal variation in *Bos taurus*. Biol Lett. 2008;4: 752-754.

56. Götherström A, Anderung C, Hellborg L, Elburg R, Smith C, Bradley DG, et al. Cattle domestication in the Near East was followed by hybridization with aurochs bulls in Europe. Proc Biol Sci. 2005;272: 2345-2350.

57. Edwards CJ, Ginja C, Kantanen J, Pérez-Pardal L, Tresset A, Stock F, et al. Dual origins of dairy cattle farming - evidence from a comprehensive survey of European Y-chromosomal variation. PLoS One. 2011 Jan 06. doi: 10.1371/journal.pone.0015922

58. Svensson EM, Häsler S, Nussbaumer M, Rehazek A, Omrak A, et al. (2014) Medieval cattle from Bern (Switzerland): An archaeozoological, genetic and historical approach. Schweiz Arch Tierheilkd 156: 17-26.

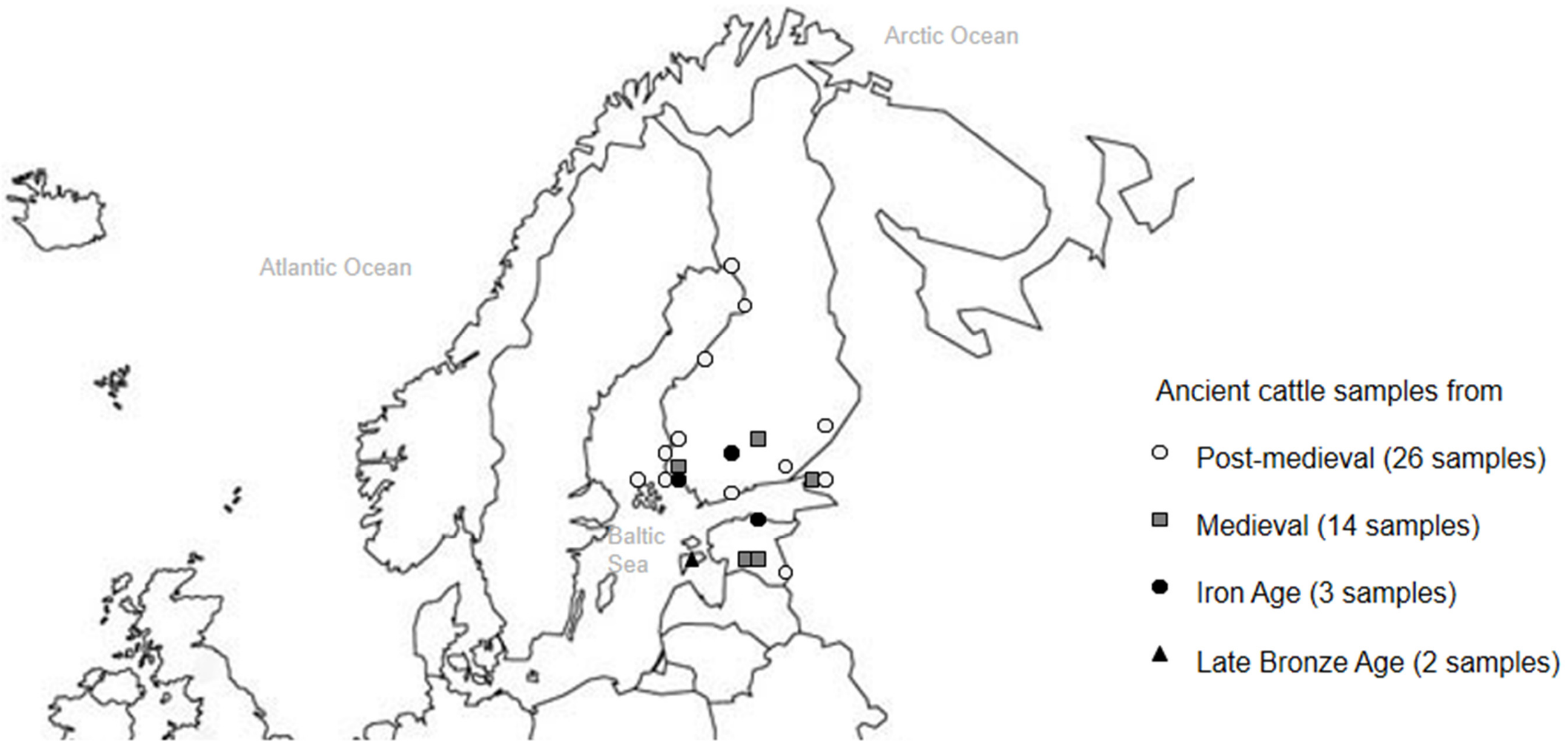

Fig. A. Excavation sites for Ancient cattle samples

Excavation sites for Ancient cattle samples in Finland, Estonia, and Vyborg in Western Russia from the Late Bronze Age (in black triangle), Late Iron Age (in black dot), Medieval (in grey square), and Post-Medieval (in white dot) periods are indicated; only the excavation sites of the samples included in the statistical analyses are shown (for more information see the main text).

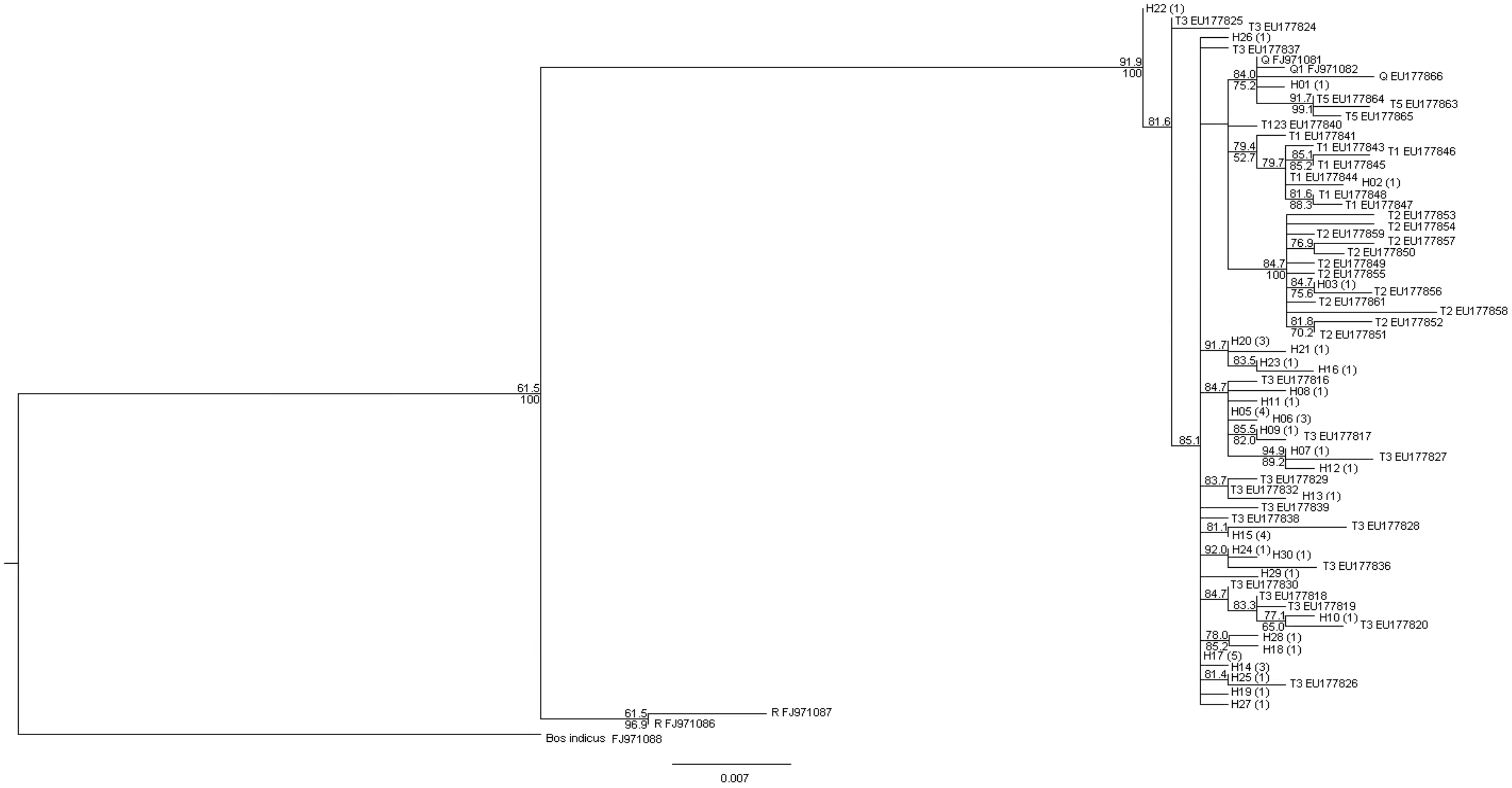

Fig. B. Maximum Likelihood tree of mtDNA haplotype sequences found within ancient cattle and modern reference sequences.

Branch topography supported by bootstrap values (on top of the branch) and Bayesian posterior probabilities (under the branch) greater than 50% are indicated. The number of ancient samples sharing the haplotype is given in parentheses. Major haplogroups (T1, T2, T3, T5, Q and R) are defined by inclusion of 43 modern reference haplotypes. The tree is rooted with a sequence of zebu (*Bos indicus*).

11	1	1111111111111111111111111111			
33	5	6666666666666666666666666666			
00	9	000000000111111111222222223 3 1111			
03	5	445557899011133489034455690000677			
58	3	290784323803935156017805091286934	Haplotype	frequency	Haplogroup
AG	C	TCCGCTAAGTCTTTTTGGGCCCATCACGGTAAC	V00654		
G.	G	.................A.....C......G..	H01	1	Q
..	*na*	..T....................C...A..G..	H02	1	T1f
..	*na*	...C............A......C......GG.	H03	1	T2
??	*na*	????????????????A......C......G..	H04	1	T2
..	*na*	....	H05	4	T3b
..	*na*	...............................G.	H06	3	T3b
..	*na*	.....C................G..........	H07	1	T3b
..	*na*	...........C.................C...	H08	1	T3b
..	*na*	...............C.................	H09	1	T3b
..	*na*	C.....G........................G.	H10	1	T3b
..	*na*	...........................A.....	H11	1	T3b
..	*na*	.....C................G.........T	H12	1	T3b
..	*na*	.............C.......T...........	H13	1	T3b
..	*na*	.......G......................G..	H14	3	T3
..	*na*	............C.................G..	H15	4	T3
..	*na*	.....C....T.............T....CG..	H16	1	T3
..	*na*	..............................G..	H17	5	T3
..	*na*	.....C........C...............G..	H18	1	T3
..	*na*	........A.....................G..	H19	1	T3
..	*na*	..........T...................G..	H20	3	T3
..	*na*	.........CT...............T...G..	H21	1	T3
..	*na*	...A..................G.......G..	H22	1	T3
..	*na*	.....C....T...................G..	H23	1	T3
..	*na*	............................A.G..	H24	1	T3
..	*na*	....T.........................G..	H25	1	T3
..	*na*	.........................G....G..	H26	1	T3
..	*na*	..................A...........G..	H27	1	T3
..	*na*	..............C....T..........G..	H28	1	T3
.A	*na*	....................T.........G..	H29	1	T3
..	*na*	.T..........................A.G..	H30	1	T3
na	*na*	C..........C....?????????????..G.	NA	1	NA
na	*na*	..........T.....?????????????????	NA	1	NA
na	*na*	????????????????....T.........???	NA	1	NA
na	*na*	?????????????????????????????.G..	NA	1	NA

Fig. C. mtDNA haplotypes found in 31 Finnish, 8 Russian and 7 Estonian ancient cattle.

Vertical numbers indicate the SNP positions relative to the reference sequence [GenBank:V00654]. Sequences were amplified from 12 911 to 13 091 and from 16031 to 178. Only the variable sites are indicated. A dot (.) indicates base similar to reference sequence. A question mark indicates a position not amplified. NA indicates position, haplotypes and haplogroups not analysed. Thirty haplotypes are indicated as H01-H30. Finally the frequency of each haplotype is given.

Table A. Ancient samples studied in this article.

Identity codes used in present aDNA analysis (Sample ID) and radiocarbon analyses (Hela-codes), archaeological site (Site), town (Location) where samples were excavated and museum ID, bone type, and sex according to osteological analysis. Radiocarbon date (Radiocarbon date BP (± 1σ)) and calibrated date (with confidence interval of 95%) or dating by the context (DBC) and corresponding historical period (Dating). Only unclear contexts were radiocarbon dated (see text for details). Success rate in sequence analysis of the mtDNA D-loop (mtDNA) and Y-chromosomal *UTY19* marker (*UTY19*). Total number of extractions (N extracts), PCR reactions (PCR), amplicons (N amplicons), and aDNA laboratories (aDNA laboratory) for each sample. Samples not analysed (-).

SAMPLE						DATING		aDNA					
**Sample ID**	**Location**	**Site**	**Museum ID***	**Bone type**	**Sex**	**Radiocarbon date**	**Dating**	**mtDNA**	***UTY19***	**N extracts**	**PCR**	**N amplicons**	**aDNA laboratory****
BtAA1	Turku, Finland	Åbo Akademi [11]	TMM 21816:14	Metacarpal	Female	DBC	Post-Medieval	Yes	No	2	15	20	SU, MTT, HU
BtAA2	Turku, Finland	Åbo Akademi [11]	TMM 21816:14	Metacarpal	Probably Female	DBC	Post-Medieval	Yes	No	2	13	20	SU, MTT, HU
BtAA3	Turku, Finland	Åbo Akademi [11]	TMM 21816:104	Metacarpal	Female	DBC	Medieval	Yes	No	3	23	20	SU, MTT, HU
BtAA4	Turku, Finland	Åbo Akademi [11]	TMM 21816:104	Metacarpal	Male	DBC	Medieval	Yes	Yes, Y2	3	26	27	SU, MTT, HU
BtAA5, Hela-2590	Turku, Finland	Åbo Akademi [11]	TMM 21816:513B	Metacarpal	Female	606 ± 30 BP, 1296 -1406 cal AD	Medieval	Yes	No	3	17	25	SU, MTT, HU
BtAA6, Hela-2589	Turku, Finland	Åbo Akademi [11]	TMM 21816:513B	Metacarpal	Female	740 ± 30 BP, 1223-1291 cal AD	Medieval	Yes	No	2	17	19	SU, MTT, HU
BtHel1	Helsinki, Finland	Snellmaninkatu [12]	KM2000002: 2174	Metacarpal	Female	DBC	Post-Medieval	Yes	No	3	17	25	SU, MTT, HU
BtHel2	Helsinki, Finland	Snellmaninkatu [12]	KM2000002: 2229	Metacarpal	Female	DBC	Post-Medieval	Yes	No	2	14	20	SU, MTT, HU
BtM2, Hela-2546	Raisio, Finland	Mulli [13]	TYA^{MR} 631:410a	Metatarsal		984 ± 30 BP, 991-1058 cal AD (50.1%), 1076-1155cal AD(45.3%)	Iron Age	Yes	No	4	18	26	SU, MTT, HU
BtMik1, Hela-2550	Mikkeli, Finland	Moisio Latokartano [14]	KM 28014:38	Tooth		567 ± 30 BP, 1305-1365cal AD(55.4%), 1384-1425 cal AD(40.0%)	Medieval (context: Iron)	Yes	No	2	13	17	SU, MTT, HU
BtNaa1, Hela-2580	Naantali, Finland	Luostari [15]	KM 2005034:196 context 986	Metacarpal		431 ± 30 BP, 1420-1498cal AD (91.7%), 1601-1615cal AD (3.7%)	Medieval	Yes	No	3	17	26	SU, MTT, HU
BtOul1	Oulu, Finland	Kajaaninkatu [16]	KM 20004081:78	Calcaneus		DBC	Post-Medieval	Yes	Yes, Y2	3	36	24	SU, MTT, HU
BtOul2	Oulu, Finland	Lyseo [17]	KM 2007031:373	Radius		DBC	Post-Medieval	Yes	No	3	26	15	SU, MTT, HU
BtPie1	Pietarsaari, Finland	Lassfolk [18]	KM 2008051:398	Metacarpal	Male	DBC	Post-Medieval	Yes	Yes, Y2	3	36	24	SU, MTT, HU
BtPie2	Pietarsaari, Finland	Lassfolk [18]	KM 2008051:3987	Metacarpal	Unsure	DBC	Post-Medieval	Yes	Yes, Y1	3	26	28	SU, MTT, HU

SAMPLE						DATING		aDNA					
**Sample ID**	**Location**	**Site**	**Museum ID***	**Bone type**	**Sex**	**Radiocarbon date**	**Dating**	**mtDNA**	***UTY19***	**N extracts**	**PCR**	**N amplicons**	**aDNA laboratory****
BtSys1, Hela-2549	Sysmä, Finland	Ihananiemi [19]	KM 32291:552	Talus		637 ± 30 BP, 1284-1330cal AD (40.5%), 1340-1397cal AD (54.9%)	Medieval	Yes	unreadable	3	27	18	SU, MTT, HU
BtTor1	Tornio, Finland	Keskikatu [20]	KM 2002081: SY7010	Metacarpal	Female	DBC	Post-Medieval	Yes	No	3	13	22	SU, MTT, HU
BtTor2	Tornio, Finland	Keskikatu [20]	KM 2002081: SY7010	Metacarpal	Female	DBC	Post-Medieval	Yes	No	2	16	19	SU, MTT, HU
BtUuk1, Hela-2583	Uukuniemi, Finland	Papinniemi [21]	KM 2001058:187	Metacarpal		238 ± 30 BP, 1527-1554 cal AD (4.4%), 1632-1682 cal AD (50%), 1738-1753 cal AD (1.8%), 1762-1803 cal AD (30.6%), 1937-1955 cal AD (8.6%)	Post-Medieval	Yes	unreadable	3	28	29	SU, MTT, HU
BtUuk3, Hela-2581	Uukuniemi, Finland	Papinniemi [21]	KM 98040:413	Metacarpal	Female	350 ± 30 BP, 1457-1635cal AD	Post-Medieval	Yes	unreadable	2	18	23	SU, MTT, HU
BtHam2	Hamina, Finland	Kortteli 23 (kasarmi) [22]	KM 20008042:170	Humerus		DBC	Post-Medieval	Yes	No	3	16	20	MTT, HU
BtHame1, Hela-2690	Hämeenlinna Finland	Varikkoniemi [23]	KM 27424:1254	Pd 4 max		985 ± 30 BP, 990-1060cal AD (51.2%), 1075-1155 cal AD (44.2%)	Iron Age	Yes	No	2	13	22	MTT, HU
BtKok1, 2592	Kökar, Åland, Finland	Kloster [24]	ÅM 640	Metatarsal		324 ± 30 BP, 1479-1645cal AD	Post-Medieval	Yes	No	3	13	18	MTT, HU
BtKok2, Hela-2591	Kökar, Åland, Finland	Kloster [24]	ÅM 640	Metatarsal		384 ± 30 BP, 1443-1524cal AD (64.8%), 1558-1631cal AD (30.6%)	Post-Medieval	Yes	No	3	15	21	MTT, HU
BtKok3	Kökar, Åland, Finland	Kloster [24]	ÅM 640	Metatarsal		DBC	Post-Medieval	Yes	Yes, Y2	3	17	25	MTT, HU
BtKok4	Kökar, Åland, Finland	Kloster [24]	ÅM 677:542	Metatarsal		DBC	Post-Medieval	Yes	No	3	26	15	MTT, HU
BtPin10	Turku, Finland	Pinella [25]	TMM 22600: M252, 216	Ct		DBC	Medieval	Partial	No	3	22	8	MTT
BtTor3	Tornio, Finland	YIT [26]	KM 2010045: 1426	Metacarpal	Female	DBC	Post-Medieval	Yes	No	2	16	20	MTT, HU
BtTor4	Tornio, Finland	YIT [26]	KM 2010045: 1475	Metacarpal	Female	DBC	Post-Medieval	Yes	No	3	26	32	MTT, HU
BtTor5	Tornio, Finland	YIT [26]	KM 2010045:1689	Metacarpal		DBC	Post-Medieval	Yes	Yes, Y2	2	21	15	MTT
BtTor6	Tornio, Finland	YIT [26]	KM 2010045:1689	Metacarpal		DBC	Post-Medieval	Yes	No	2	17	19	MTT, HU
BtAsv1	Asva, Island of Saaremaa, Estonia	Settlement site [27]	AI 4366	Metatarsal		DBC	Late Bronze Age	Yes	No	2	24	24	MTT, HU
BtOrd1	Viljandi, Estonia,	Order castle [28]	VM 10922	Tooth, Molar		DBC	Medieval	Yes	No	2	12	18	MTT, HU
BtOte1	Otepää, Estonia	Hillfort [29]	AI 4036	Mandible		DBC	Medieval	Yes	No	2	12	18	MTT, HU

SAMPLE						DATING		aDNA					
**Sample ID**	**Location**	**Site**	**Museum ID***	**Bone type**	**Sex**	**Radiocarbon date**	**Dating**	**mtDNA**	***UTY19***	**N extracts**	**PCR**	**N amplicons**	**aDNA laboratory****
BtPad1	Pada, Estonia	Settlement site [30]	AI 5200	Tooth, Molar		DBC	Iron Age	Yes	No	3	19	25	MTT, HU
BtRid1	Ridala, Island of Saaremaa, Estonia	Fortified settlement site [27]	AI 4261	Metatarsal		DBC	Late Bronze Age	Yes	No	2	12	20	MTT, HU
BtSoon1	Soontagana, Estonia	Hillfort [31]	PäMu 2 / A 2434	Metacarpal		DBC	Iron Age	Unrepeatable	No	3	20	19	MTT, HU
BtVast1	Vastseliina, Estonia	Castle [32]	TÜ 1435	Metatarsal		DBC	Post-Medieval	Yes	No	2	14	24	MTT, HU
BtVast2	Vastseliina, Estonia	Castle [32]	TÜ 1499	Tooth, Molar		DBC	Post-Medieval	Yes	No	3	12	20	MTT, HU
BtViip1	Vyborg, Russia	Vyborg [33]	RAS, Level 13	Metatarsal		DBC	Medieval	Yes	No	2	12	15	MTT, HU
BtViip2	Vyborg, Russia	Vyborg [33]	RAS, Level 14	Metatarsal		DBC	Medieval	Yes	No	2	13	15	MTT, HU
BtViip3	Vyborg, Russia	Vyborg [33]	RAS, Level 14	Metatarsal		DBC	Medieval	Yes	No	2	12	16	MTT, HU
BtViip4	Vyborg, Russia	Vyborg [33]	RAS, Level 13	Metatarsal		DBC	Medieval	Yes	Yes, Y2	3	28	32	MTT, HU
BtViip5	Vyborg, Russia	Vyborg [33]	RAS, Level 13	Metatarsal		DBC	Medieval	Yes	No	3	15	22	MTT, HU
BtViip6	Vyborg, Russia	Vyborg [33]	RAS, Level 2	Metatarsal		DBC	Post-Medieval	Yes	No	3	11	20	MTT, HU
BtViip7	Vyborg, Russia	Vyborg [33]	RAS, Level 2	Metatarsal		DBC	Post-Medieval	Yes	No	3	16	18	MTT, HU
BtViip8	Vyborg, Russia	Vyborg [33]	RAS, Level 2	Metatarsal		DBC	Post-Medieval	Yes	No	2	13	16	MTT, HU
BtLui1	Eura, Finland	Luistari [34]	KM 22346:461, 462 (grave 494)	Tooth		DBC	Iron Age	No	No	2	9	0	SU
BtM1, Hela-2545	Raisio, Finland	Mulli [13]	TYA^{MR} 619:903a	Metatarsal		990 ± 30 BP, 988-1054 cal AD (57.0%), 1079-1154 cal AD (38.4%)	Iron Age	No	No	2	7	0	SU
BtM3, Hela-2547	Raisio, Finland	Mulli [13]	TYA^{MR} 619:950a	Metatarsal		953 ± 30 BP, 1023 1155 cal AD	Iron Age	No	No	2	9	0	SU
BtM4, Hela-2548	Raisio, Finland	Mulli [13]	TYA^{MR} 619:864d	Metatarsal		990 ± 30 BP, 988-1054 cal AD (57.0%), 1079-1154 cal AD (38.4%)	Iron Age	No	No	2	9	0	SU
BtOul3	Oulu, Finland	Pikisaari [35]	KM 2006047:62, SY 5	Calcaneus		DBC	Post-Medieval	No	No	2	9	0	SU
BtPih1, Hela-2579	Pihtipudas, Finland	Hämeensaari [36]	KM 27198:39	Tibia		323 ± 30 BP, 1479-1645 cal AD	Post-Medieval (context: Iron)	No	No	2	9	0	SU
BtUuk2, Hela-2582	Uukuniemi, Finland	Papinniemi [21]	KM 2001058:189	Metacarpal		NA, DBC	Post-Medieval	No	No	2	9	0	SU
BtHir1,	Hirvensalmi,	Vahvajärvi, Lampuunlahti	No ID, collected	Rib		363 ± 30 BP,	Post-	No	No	2	9	0	MTT

SAMPLE						DATING		aDNA					
**Sample ID**	**Location**	**Site**	**Museum ID***	**Bone type**	**Sex**	**Radiocarbon date**	**Dating**	**mtDNA**	***UTY19***	**N extracts**	**PCR**	**N amplicons**	**aDNA laboratory****
Hela-2588	Finland	[37]	from surface			1449-1529 cal AD (50.1%), 1544-1634 cal AD (45.3%)	Medieval (context: Medieval)						
BtLie1, Hela-2587	Lieto, Finland	Aittamäki [38]	TYA 597:102	Metatarsal		1814 ± 44 BP, 84-263 cal AD (83.8%), 277-330 cal AD (11.6%)	Iron Age	No	No	2	9	0	MTT
BtVes1, Hela-2578	Vesilahti, Finland	Hinsala, Tonttimäki [39]	TYA 335:330	Phalanx 2		MODERN	Modern (context: Iron)	No	No	1	11	0	MTT
BtVar2	Helsinki, Finland	Vartiokylä [40]	KM 33374:1010	Metatarsal		DBC	Medieval	No	No	1	8	0	MTT
BtEno1	Enontekiö, Finland	Markkina [41]	KM 32131:1025	Humerus		DBC	Post-Medieval	No	No	1	8	0	MTT
BtVar1	Helsinki, Finland	Vartiokylä [40]	KM 33374:1018	Metatarsal		DBC	Medieval	No	-	2	8	0	MTT
BtMik2	Mikkeli, Finland	Porrassalmenpelto [42]	KM 10629: 50	Pd4 mand		DBC	Iron Age	No	-	2	8	0	MTT
BtMik4	Mikkeli, Finland	Porrassalmenpelto [42]	KM 10629:70	Phalanx 1		DBC	Iron Age	No	-	2	8	0	MTT
BtPin11	Turku, Finland	Pinella [25]	TMM 22600: M252, 217	Ct		DBC	Medieval	No	-	2	8	0	MTT
BtAbo18	Turku, Finland	Aboa Vetus [43]	KM^{AV}2010001:718 1139 luode 2	Mandible		DBC	Medieval	Partial	No	2	9	4	MTT
BtHäm2	Hämeenlinna, Finland	Varikkoniemi [23]	KM27424: 1254	Phalanx 1		DBC	Iron Age / Early Medieval	No	-	2	7	0	MTT
BtSul1	Sulkava, Finland	Keskipelto [44]	KM27658:8	Pars petrosa		DBC	Iron Age	No	-	1	7	0	MTT
BtPin4	Turku, Finland	Pinella [25]	TMM 22600: M215, 143	Metacarpal	Male	DBC	Medieval	No	-	2	7	0	MTT
BtPin8	Turku, Finland	Pinella [25]	TMM 22600: M240, 168	Metacarpal		DBC	Medieval	No	-	2	7	0	MTT
BtHam1	Hamina, Finland	Kortteli 23 (kasarmi) [22]	KM 20008042: 170	Humerus		DBC	Post-Medieval	No	No	1	10	0	MTT
BtPih2	Pihtipudas, Finland	Niemi [45]	KM27198:39	Phalanx 1		DBC	Post-Medieval	No	No	1	9	0	MTT
BtSas1	Sastamala, Finland	Vehmaan kylätontti [46]	KM 2009094	Metacarpal		DBC	Post-Medieval	No	No	1	10	0	MTT
BtSal1	Salme, Island of Saaremaa, Estonia	Boat burial (Salme1) [47]	SM 10601	Humerus		DBC	Iron Age, Pre-Viking Age	Partial	No	1	10	4	MTT
BtJaa1	Viljandi, Estonia	St John's Church [48]	VM 10258	Tooth, Molar		DBC	Medieval	Partial	No	1	10	2	MTT
BtKiv1	Viljandi, Estonia	Kivimägi [49]	VM 10742	Tooth, Molar		DBC	Iron Age	No	No	1	10	0	MTT
BtVilm1	Viljandi,	Settlement site	VM 10847	Tooth, Molar		DBC	Iron Age	Partial	No	1	10	4	MTT

SAMPLE						DATING		aDNA					
**Sample ID**	**Location**	**Site**	**Museum ID***	**Bone type**	**Sex**	**Radiocarbon date**	**Dating**	**mtDNA**	***UTY19***	**N extracts**	**PCR**	**N amplicons**	**aDNA laboratory****
	Estonia	[50]											
BtLos1	Viljandi, Estonia	Lossi street [51]	VM 10848	Metatarsal		DBC	Medieval	Partial	No	1	10	6	MTT
BtSuu1	Viljandi, Estonia	Ski-jumping hill [52]	VM 10877	Tooth, Molar		DBC	Iron Age	No	No	2	10	0	MTT

*Museums abbreviations: AI = Institute of History, Tallinn University, Estonia, VM = Museum of Viljandi, Estonia, PäMu = Pärnu Museum, Estonia, TÜ = University of Tartu, Estonia, SM = Saaremaa Museum, Estonia, RAS = Institute of History of Material Culture, Russian Academy of Sciences, St Petersburg, Russia, KM = The National Board of Antiquities, Finland, KM^{AV} = Samples are held at the Aboa Vetus Museum, Turku, Finland, ÅM = Ålands Museum, Finland, TMM = The Museum Centre of Turku, Finland, TYA = Archives of Department of Archaeology, University of Turku, Finland, TYA^{MR} = Samples are held at the Museum of Raisio (Harkko), Finland

**Laboratories participating in aDNA analyses: SU= Stockholm University, Sweden, MTT= Agrifood research Finland, Finland, and HU= Helsinki University, Finland.

Table B. Summary of primers.

Primers, annealing temperatures (AT), fragment lengths, nucleotide positions for amplification start and average amplification success in aDNA analysis in 77 ancient samples. Nucleotide positions showing the mtDNA and *UTY19* gene amplifications are given according to sequences of [GenBank:V00654] and [GenBank:AY936543], respectively.

Fragment	Primer	AT	Fragment length	Start Position (according to V00654)	Average amplification success
D-loop fragment 1	For 5'CATTAAATTATATGCCCCATGC 3'	58	188	16 009	52 %
	Rev 5' CTAGCGGGTTGCTGGTTTC 3'			16 178	
D-loop fragment 2	For 5' TCACGAGCTTAATTACCATGC 3'	60	219	16 152	57 %
	Rev 5' TATGTGTGAGCATGGGCTGA 3'			13	
D-loop fragment 3	For 5' AGACATCTCGATGGACTAATGG 3'	60	212	16 325	56 %
	Rev 5' TGTCCTGTGACCATTGACTG 3'			179	
D-loop SNP T5	For 5'CCGTTTCTATCCCCTCACAG3'	55	221	12 891	66 %
	Rev 5'AAAAGGCGTGGGTACAGATG3'			13 092	
Bos Taurus UTY19	For 5'AGCTCCAGAATATTTCACTGACCT3'	55	155	312 (in AY936543)	*
	Rev 5'GAAGGCAAATGCAGAGACAA3'			442 (in AY936543)	

* amplification success not indicated, as the sex of all of the samples were unknown

Table C. Distribution of ancient mtDNA haplotypes found in the present and previous studies.
Haplotypes shared between ancient cattle data, from this study, and the same region (245 bp) available in previous studies. The total number of samples in each study is shown (N). The distribution of the haplotypes is also presented in Fig.1; where the remaining, unshared haplotypes are shown in white (other haplotypes). Analyses of modern data can be found following the references given in the right column.

	N	H05 H06 H11 H17 H24 H26	H09	H10	H14	H20	H25	H30	H07 H12	H01	H03	H22	H19	H23	H29	H15	H27	H16	Reference
**North-East Baltic Sea Region**																			
Prehistory	5	2																	This study
Medieval	14	4		1	1	2					1					3	1		This study
Post Medieval	26	9	1		2	1	1	1	2	1		1	1	1	1	1		1	This study
Northern Finncattle	8	4										1				1			[4]
Western Finncattle	7	3						2					1						[4]
Eastern Finncattle	25	18	1		1							2							[4,53]
Estonian Red	5	2				1													[4]
Estonian Native	4	4																	[4]
**Scandinavia**																			
Jutland breed	9	4				1												1	[4]
Danish Red	9	3				6													[4]
Blacksided Trondheim	5								3										[53]
Telemark	5	3																	[53]
Vestland Red Polled	5	2																	[53]
Swedish Red-and-White	5	2																	[4]
Fjall Cattle	6	2																	[4]
Swedish Mountain Cattle	4	2					1												[4]
Swedish Red Polled	15	5				1										2			[4,53]
Ringamala Cattle	7					6													[4]
Bohus Poll	6					6													[4]

	N	H05 H06 H11 H17 H24 H26	H09	H10	H14	H20	H25	H30	H07 H12	H01	H03	H22	H19	H23	H29	H15	H27	H16	Reference
Vane Cattle	5																		[4]
**West Europe**																			
Faroe Islands Cattle	14	11																	[4]
Swedish Holstein-Friesian	5					2													[4]
Finnish Holstein-Friesian	7	5																	[4]
Finnish Ayrshire	7	5			1														[4]
Jersey	18	7							4										[53]
Italian Friesian	190	65	1						7						1		1		[2,53]
Holstein	2																		[53]
Estonian Holstein-Friesian	1																		[4]
**South Europe**																			
Simmental	9	1																	[53]
Swiss Brown	1	1																	[53]
Limousine	53	11	1																[5,53]
Betizuak	2	1																	[2]
Agerolese	37	2																	[5,53]
Burlina	1	1																	[53]
Cabannina	43	14					2												[2,53]
Calvana	29	15																	[5,53]
Chianina	323	69			2											1			[2,5,53]
Cinisara	78	13															3		[2,5,53]
Grey Alpine	45	13													1				[53]
Grey Steppe	19	2																	[5,53]
Italian Brown	10	2					1								1				[5,53]
Italian Podolian	84	22					2									1	1		[5,53]
Italian Red Pied	126	31	2	1	2				1										[5,53]

	N	H05<br>H06<br>H11<br>H17<br>H24<br>H26	H09	H10	H14	H20	H25	H30	H07<br>H12	H01	H03	H22	H19	H23	H29	H15	H27	H16	Reference
Marchigiana	150	30											1			5			[5,53]
Maremmana	28	5					1									2			[2,5,53]
Modicana	14	9																	[2,5,53]
Mucca Pisana	33	2																	[53]
Ottonese	7	3																	[53]
Pettiazza	36	15																	[2,53]
Piedmontese	72	23				3	1		4										[2,53]
Reggiana	40	4				1	2												[5,53]
Rendena	2										1								[2,53]
Romagnola	231	36				1										1	3		[5,53]
Valdostana	54	9	1		1		1												[2,53]
Bianca Val Padana	4																		[53]
Garfagnina	2																		[53]
Savoiarda	2																		[53]
Podolica	2																		[3]
**South-East Europe**																			
Busa	8	2																	[4]
Bulgarian Grey	30	2			7														[53]
Podolian	11	4																	[4]
Greek	1																		[2]
**East Europe**																			
Byelorussian Red	8	4																	[4]
Ukrainian Grey	8	8																	[4]
Ukrainian Whitehead	10	1									2								[4]
**West Russia**																			
Kholmogory	14	8			1			4	1										[4]

	N	H05<br>H06<br>H11<br>H17<br>H24<br>H26	H09	H10	H14	H20	H25	H30	H07<br>H12	H01	H03	H22	H19	H23	H29	H15	H27	H16	Reference
Yaroslavl	10	8								2									[4]
Red Gorbatov	2					1													[4]
**Near East and Central Asia**																			
Ala-Tau	5	2															1		[4]
Iraqi	9	1																	[2]
Iranian	7	1																	[2]
Bushuev	4																		[4]
**Middle Russia**																			
Pechora type	9	6			2														[4]
Yurino	4	1				2													[4]
Suksun	10	1			2	1		1							1				[4]
Istoben	9	4												1					[4]
**Siberia**																			
Yakutian Cattle	24	14														2			[4]
**Total**	2139	563	7	2	22	35	12	8	22	3	4	4	3	2	5	19	10	2	

Table D. Fennoscandian bulls included in temporal analyses.
Y-chromosomal *UTY19* haplotype (Y1 and Y2) and number of Finnish and Swedish Iron Age, Medieval, and Post-Medieval period, and modern native bulls included in the statistical analyses. The references to original articles are given at right.

		Y1	Y2	Total	Reference
Iron Age	Sweden	1	7		[54]
	**Total**	**1**	**7**	**8**	
Medieval	Finland	0	2		This study
	Sweden	1	34		[54,55]
	**Total**	**1**	**36**	**37**	
Post-Medieval	Finland	1	4		This study
	Sweden	8	6		[55]
	**Total**	**9**	**10**	**19**	
Modern native breeds	Finland	20	8		[56] and [4] as reported in [57]*
	Sweden	13	0		[56] and [4] as reported in [57]
	**Total**	**33**	**8**	**41**	
**Total**		**44**	**61**	**105**	

*In Kantanen et al. [4] 11 (out of 28) of the Finnish bulls were also sampled and analysed in Götherström et al. [56].

Table E. Distribution of ancient and modern Eurasian Y-haplotypes.
Y-chromosomal haplotype (Y1 and Y2) in 127 modern Eurasian breeds and five ancient cohorts divided into nine geographical regions. The number of samples (N) in each breed or ancient cohort, count, and proportion of Y1 and Y2 are given. The references to original articles are given at right.

Region	Breed/population	N	Y1	Y2	Reference
**East Europe**					
	Black and White	10	10 (100 %)		[55]
	Commercial Red	10	9 (90 %)	1 (10 %)	[55]
	Latvian Blue (native)	9	9 (100 %)		[57]
	Latvian Brown (commercial)	8	8 (100 %)		[57]
	Light Grey	6	6 (100 %)		[55]
	Ukrainian Grey	5		5 (100 %)	[57]
	Ukrainian Red Steppe	5	5 (100 %)		[57]
	Ukrainian Whiteheaded	11	5 (45 %)	6 (55 %)	[57]
	White Bached	1	1 (100 %)		[55]
	Total	65	53 (82 %)	12 (18 %)	
**Central Russia**					
	Bestuzhev	4	4 (100 %)		[57]
	Istobenskaya	9	9 (100 %)		[57]
	Pechorskaya	7	7 (100 %)		[57]
	Suksunskaya	5	4 (80 %)	1 (20 %)	[57]
	Total	25	24 (96 %)	1 (4 %)	
**Near-East & Central Asia**					
	Anatolian Black	5		5 (100 %)	[56]
	Damascus	3	1 (33 %)	2 (67 %)	[57]
	East Anatolian Red	4		4 (100 %)	[56]
	Kalmyk	12		12 (100 %)	[57]
	South Anatolian Red	5		5 (100 %)	[56]
	Turkish Grey	3		3 (100 %)	[57]
	Total	32	1 (3 %)	31 (97 %)	
**Nordic countries**					
	Ancient Finnish cattle	7	1 (14 %)	6 (86 %)	This study
	Ancient Swedish cattle	57	10 (18 %)	47 (82 %)	[54,55]
	Blacksided Troender	7	7 (100 %)		[57]
	Danish Red	26	21 (81 %)	5 (19 %)	[57]
	Doela	4		4 (100 %)	[57]
	Eastern Finncattle	9	1 (11 %)	8 (89 %)	[56,57]
	Eastern Red Polled	5	5 (100 %)		[57]
	Fjallnara	3	3 (100 %)		[57]
	Icelandic	8	8 (100 %)		[57]
	Jutland (old native)	6	6 (100 %)		[57]
	Northern Finncattle	3	3 (100 %)		[56]
	Norwegian (commercial, hybrid)	12	12 (100 %)		[57]
	Swedish Mountain Cattle	10	10 (100 %)		[56,57]
	Swedish Red	2	2 (100 %)		[56]

Region	Breed/population	N	Y1	Y2	Reference
	Swedish Red Polled	5	5 (100 %)		[56,57]
	Telemark	2		2 (100 %)	[57]
	Western Finncattle	9	9 (100 %)		[56,57]
	Western Fjord	6	6 (100 %)		[57]
	Western Red Polled	3	3 (100 %)		[57]
	Total	184	112 (61 %)	72 (39 %)	
**Russian Siberia**					
	Yakutian cattle	23		23 (100 %)	[57]
	Total	23		23 (100 %)	
**South-East Europe**					
	Ancient Hungary	1		1 (100 %)	[55]
	Busha	5		5 (100 %)	[57]
	Serbian Podolica	4		4 (100 %)	[57]
	Total	10		10 (100 %)	
**South and Central Europe**					
	Ancient Bern	14	1 (7 %)	13 (93 %)	[58]
	Alentejana	36		36 (100 %)	[57]
	Alistana-Sanabresa	12		12 (100 %)	[57]
	Arouquesa	33		33 (100 %)	[57]
	Asturiana de los Valles	38	32 (84 %)	6 (16 %)	[57]
	Asturiana de Montana	19	18 (95 %)	1 (5 %)	[57]
	Avilena Negro Iberica	7	1 (14 %)	6 (86 %)	[57]
	Barrosã	33		33 (100 %)	[57]
	Berrenda	5		5 (100 %)	[57]
	Betizu	17		17 (100 %)	[57]
	Blonde d'Aquitaine	7		7 (100 %)	[57]
	Braunvieh	3		3 (100 %)	[56]
	Brava de Lide	26	2 (8 %)	24 (92 %)	[57]
	Brunade los Pirineds	11		11 (100 %)	[57]
	Cabannina	2		2 (100 %)	[57]
	Cachena	25		25 (100 %)	[57]
	Charolais	37		37 (100 %)	[57]
	Chianina	22		22 (100 %)	[57]
	De Lida	2		2 (100 %)	[56]
	Ehringer	2		2 (100 %)	[56]
	Fleckvieh	3		3 (100 %)	[56]
	Garvonesa	6		6 (100 %)	[57]
	Gelbvieh	4		4 (100 %)	[56]
	Istrian	4		4 (100 %)	[57]
	Lidia	66		66 (100 %)	[57]
	Limousin	26		26 (100 %)	[57]
	Mallorquina	8	8 (100 %)		[57]
	Marchigiana	11		11 (100 %)	[57]
	Maremmana	19		19 (100 %)	[57]
	Marinhoa	17		17 (100 %)	[57]
	Maronesa	23		23 (100 %)	[57]
	Menorquina	4	4 (100 %)		[56]

Region	Breed/population	N	Y1	Y2	Reference
	Mertolenga	23	8 (35 %)	15 (65 %)	[57]
	Minhota	28		28 (100 %)	[57]
	Mirandesa	23		23 (100 %)	[57]
	Montbeliard	6		6 (100 %)	[57]
	Morucha	5		5 (100 %)	[57]
	Mostrenca	21	21 (100 %)		[57]
	Pajuna	4	1 (25 %)	3 (75 %)	[57]
	Parthenaise	15	4 (27 %)	11 (73 %)	[57]
	Piemontese	17		17 (100 %)	[57]
	Pinzgaur	13		13 (100 %)	[57]
	Pirenaica	10		10 (100 %)	[57]
	Podolica	13		13 (100 %)	[57]
	Preta	29	1 (3 %)	28 (97 %)	[57]
	Pustertaler	13		13 (100 %)	[57]
	Red Holstein	3		3 (100 %)	[57]
	Rendena	4		4 (100 %)	[56]
	Retinta	6		6 (100 %)	[57]
	Romagnola	4		4 (100 %)	[56]
	Rotbunte	4	4 (100 %)		[56]
	Rubia Gallega	44		44 (100 %)	[57]
	Salers	20	1 (5 %)	19 (95 %)	[57]
	Sayaguesa	8		8 (100 %)	[57]
	Schawarzbunte-HF	4	4 (100 %)		[56]
	Simmental	20	1 (5 %)	19 (95 %)	[57]
	Swiss Brown	14		14 (100 %)	[57]
	Tarentaise	18		18 (100 %)	[57]
	Tudanca	10	10 (100 %)		[57]
	Tyrolean Grey	19		19 (100 %)	[57]
	Total	940	121 (13 %)	819 (87 %)	
**West Europe**					
	Aberdeen Angus	37	37 (100 %)		[57]
	Angler	14	14 (100 %)		[57]
	Angus	4	3 (75 %)	1 (25 %)	[56]
	Ayrshire	22	22 (100 %)		[57]
	Belgian Blue	21	21 (100 %)		[57]
	Belgian Red	4	4 (100 %)		[57]
	British White	21	17 (81 %)	4 (19 %)	[57]
	Dexter	4	4 (100 %)		[57]
	Dutch Belted	8	3 (38 %)	5 (63 %)	[57]
	Fries	5	5 (100 %)		[56]
	Friesian	4	4 (100 %)		[56]
	Friesian-Dutch	8	8 (100 %)		[57]
	Galloway	11		11 (100 %)	[57]
	German Original Black Pied-West	3	3 (100 %)		[57]
	Groningen Whitehead	11	11 (100 %)		[57]
	Guernsey	4		4 (100 %)	[56]

Region	Breed/population	N	Y1	Y2	Reference
	Hereford	31	30 (97 %)	1 (3 %)	[57]
	Highland	13		13 (100 %)	[57]
	Holstein Friesian	65	65 (100 %)		[57]
	Jersey	28		28 (100 %)	[57]
	Lakenvalder	5	2 (40 %)	3 (60 %)	[56]
	Lowland	2	2 (100 %)		[56]
	Meuse-Rhine-Yssel	13	13 (100 %)		[57]
	Normand	46	46 (100 %)		[57]
	Red Holstein	1	1 (100 %)		[57]
	Shorthorn	19	19 (100 %)		[57]
	Total	404	334 (83 %)	70 (17 %)	
**West Russia**					
	Kholomogorskaya	6	6 (100 %)		[57]
	Yaroslavskaya	3	3 (100 %)		[57]
	Total	9	9 (100 %)		
Total data		1692	654 (39 %)	1038 (61 %)	
